# Investigating the Protonated Cannabinoid Dimer Detected in the Forensic Analysis of *Cannabis* by DART-MS: A Combined Mass Spectrometry and Computational Study

**DOI:** 10.1021/jasms.5c00433

**Published:** 2026-03-21

**Authors:** Parandaman Arathala, Benedetta Garosi, Megan I. Chambers, Robert B. Cody, Rabi A. Musah

**Affiliations:** † Department of Chemistry, 1084 Louisiana State University, Baton Rouge, Louisiana 70803, United States; ‡ Department of Chemistry, University at Albany, State University of New York, Albany, New York 12222, United States; § JEOL USA Inc., 11 Dearborn Road, Peabody, Massachusetts 01960, United States

**Keywords:** protonated cannabinoid dimer, Cannabis, forensic analysis, DART-HRMS, FD-MS, density functional theory, population analysis

## Abstract

Direct analysis in real time–high-resolution mass spectrometry (DART-HRMS) enables the rapid chemical profiling of *Cannabis* samples. This approach reveals a prominent signal at nominal *m*/*z* 315, characteristic of protonated isomeric cannabinoids such as Δ^9^-tetrahydrocannabinol (Δ^9^-THC), cannabidiol (CBD), cannabichromene (CBC), Δ^8^-THC, and cannabicitran (CBT). Interestingly, the spectra of standards of these cannabinoids also display a peak at nominal *m*/*z* 629, corresponding to the protonated dimer species [2M + H]^+^. To elucidate the likely structures of these ions, density functional theory calculations were performed for protonated homo- and heterodimer combinations of the five cannabinoids. The calculations indicated that CBDH^+^••CBD, Δ^9^-THCH^+^••Δ^9^-THC, and Δ^8^-THCH^+^••CBC are the most stable. Population analysis calculations further revealed a temperature dependence, with Δ^8^-THCH^+^••CBC being the dominant species below 240 K, while CBDH^+^••CBD becomes the most abundant in the 240–800 K range. These findings imply that at a DART gas temperature of 623 K, the peak at *m*/*z* 629 detected in hemp, where CBD levels are high, is primarily attributable to CBDH^+^••CBD. Conversely, the population analysis studies showed that marijuana, which contains negligible CBD, would not be expected to exhibit a mass at *m*/*z* 629. Experimental DART-HRMS and field desorption (FD)-MS measurements of hemp and marijuana validated this prediction. The results support the conclusion that the peak at *m*/*z* 629 specifically observed in hemp samples is representative of the CBDH^+^••CBD adduct, as opposed to a natural product molecular marker of hemp, and it provides a rapid and reliable mass by which to distinguish hemp from marijuana by DART-HRMS.

## Graphical abstract

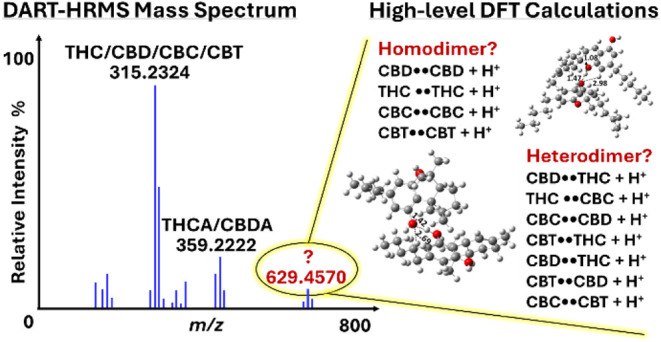

## 1 Introduction

Under the United Nations Single Convention on Narcotic Drugs treaty, plant species in the *Cannabis* genus, the most common of which is *C. sativa*, are Schedule I drugs.^1^ Despite this designation, it remains the most widely used psychoactive substance in the world. Depending on the country, penalties for its possession extend from minor (e.g., misdemeanor or noncriminal offense classifications for possession of small quantities) to the death penalty. Because the potential stakes for its sale, possession, and use are, in most cases, very high, enforcement agencies responsible for ensuring compliance with the law must have dependable methods to confirm its identity. In this regard, crime laboratories around the world have relied heavily on gas chromatography–mass spectrometry (GC-MS) analysis for the detection of its major psychoactive component, Δ^9^-tetrahydrocannabinol (Δ^9^-THC), along with observation of the characteristic trichomes (cystolithic hairs) that are observable in the plant material by light microscopy, as a means to confirm that a substance is correctly designated as *Cannabis*.

One of the factors that complicates the legislation of *Cannabis* is that there are varieties of the plant that are economically important as an agricultural commodity cultivated for industrial and commercial uses. In the United States, the federal government, through the enactment of the Agriculture Improvement Act of 2018, defined two classifications for *Cannabis*: formally, hemp is plant material that contains ≤0.3% Δ^9^-THC by dry weight, and that which contains >0.3% Δ^9^-THC by mass is marijuana.^2^ Since the former is a legal product and the latter a Schedule I substance subject to the imposition of penalties for possession, distribution, and consumption, law enforcement agencies have been burdened with the responsibility of interrogating seized materials that appear to be *Cannabis* in order to determine whether it is legal hemp or illegal marijuana. This is onerous because it is time-consuming to quantify the concentration of THC present; costly because of the amounts of consumables required for routine analysis; and labor-intensive in terms of the human resources required to perform the analyses by the well-established conventional methods. For these reasons, there are ongoing efforts to develop approaches that will enable rapid and efficient forensic differentiation of hemp and marijuana in a fashion that circumvents the aforementioned challenges. Of the alternative approaches that could be utilized, ambient ionization MS (AIMS) methods appear to be particularly well-suited, as sample preparation steps can be bypassed since materials can be analyzed in their native form, analysis is high-throughput, use of consumables is minimized, and the resultant time savings reduce the human resource hours when compared to that required using common traditional methods.

Currently, among the available AIMS methods being deployed for the analysis of *Cannabis*, direct analysis in real time–high-resolution mass spectrometry (DART-HRMS) enjoys the most widespread adoption in crime laboratories in the U.S. While it is not generally used as a *confirmatory* analysis technique (because, unlike GC-MS, it does not involve the use of a chromatographic dimension that would provide an additional sample identification measure through the use of compound retention times), it cuts down on analysis time by serving as a triage approach for rapid screening of samples to indicate whether they exhibit *m*/*z* values consistent with the presence of characteristic cannabinoids. However, its utility in crime laboratories would be enhanced if its use could be extended beyond triage to include quantification of analytes of interest, as well as the differentiation of hemp and marijuana. Recent studies have shown how DART-MS can be used for the rapid qualitative detection of phytocannabinoids in plant materials and other complex matrices such as edibles and personal care products.^3,4^ It has also been demonstrated that multivariate statistical analysis of DART-MS-derived chemical profiles can enable the classification of hemp cultivars.^5^ Another study extended the application of chemometrics to *Cannabis* DART-MS data to successfully differentiate between hemp and marijuana with 100% accuracy for the external validation samples.^6^ In that study, a subset of the *m*/*z* values that were most heavily weighted in enabling the random forest prediction model to correctly classify hemp and marijuana samples was determined, with the most important of these being a mass observed for hemp samples that is consistent with the presence of [2 x C_21_H_30_O_2_ + H]^+^ at nominal *m*/*z* 629. The formula C_21_H_30_O_2_ corresponds to Δ^9^-THC and its isomers, and therefore, *m*/*z* 629 could correspond to a protonated dimer of molecules with the formula C_21_H_30_O_2_.

In DART-MS, detection of the protonated dimers of analytes is not uncommon. For example, the protonated dimer of capsaicin, an active component in peppers with the chemical formula C_18_H_27_NO_3_, is detected in the DART-HRMS analysis of habanero peppers.^7^ DART-MS analysis of sugar alcohol precursors of nitrate ester explosives shows ammonium adduct monomers [M + NH_4_]^+^, protonated dimers [2M + H]^+^, and ammonium adduct dimers [2M + NH_4_]^+^.^8^ However, while the mass at nominal *m*/*z* 629 which is diagnostic for hemp could be a protonated dimer of C_21_H_30_O_2_, it is possible, in principle, for the mass to be representative of a condensed dimer (i.e., the protonated form of a bona fide natural product dimer molecule that is a specific marker for hemp). Recently, a class of phytocannabinoid condensed dimers has been isolated and structurally characterized by high-resolution electrospray ionization-mass spectrometry (HR-ESI-MS), GC-MS, and nuclear magnetic resonance (NMR) spectroscopy.^9,10^ They include cannabitwinol, a cannabidiol (CBD) condensed dimer^10^ (CBDCD), and cannabisol, a condensed dimer of THC (THCCD).^9^ Both are comprised of two cannabinoid units connected via a methylene bridge^9,10^ (see Figure 1). The discovery of these molecules could indicate the existence of additional cannabinoid condensed dimers or other natural products that could account for *m*/*z* 629 that is specifically observed in DART-HRMS analysis of hemp. We sought in this work to determine the identity of *m*/*z* 629 since it was a reliable marker that enabled rapid and accurate identification of plant material as hemp when it was analyzed by DART-MS.

**Figure 1:**
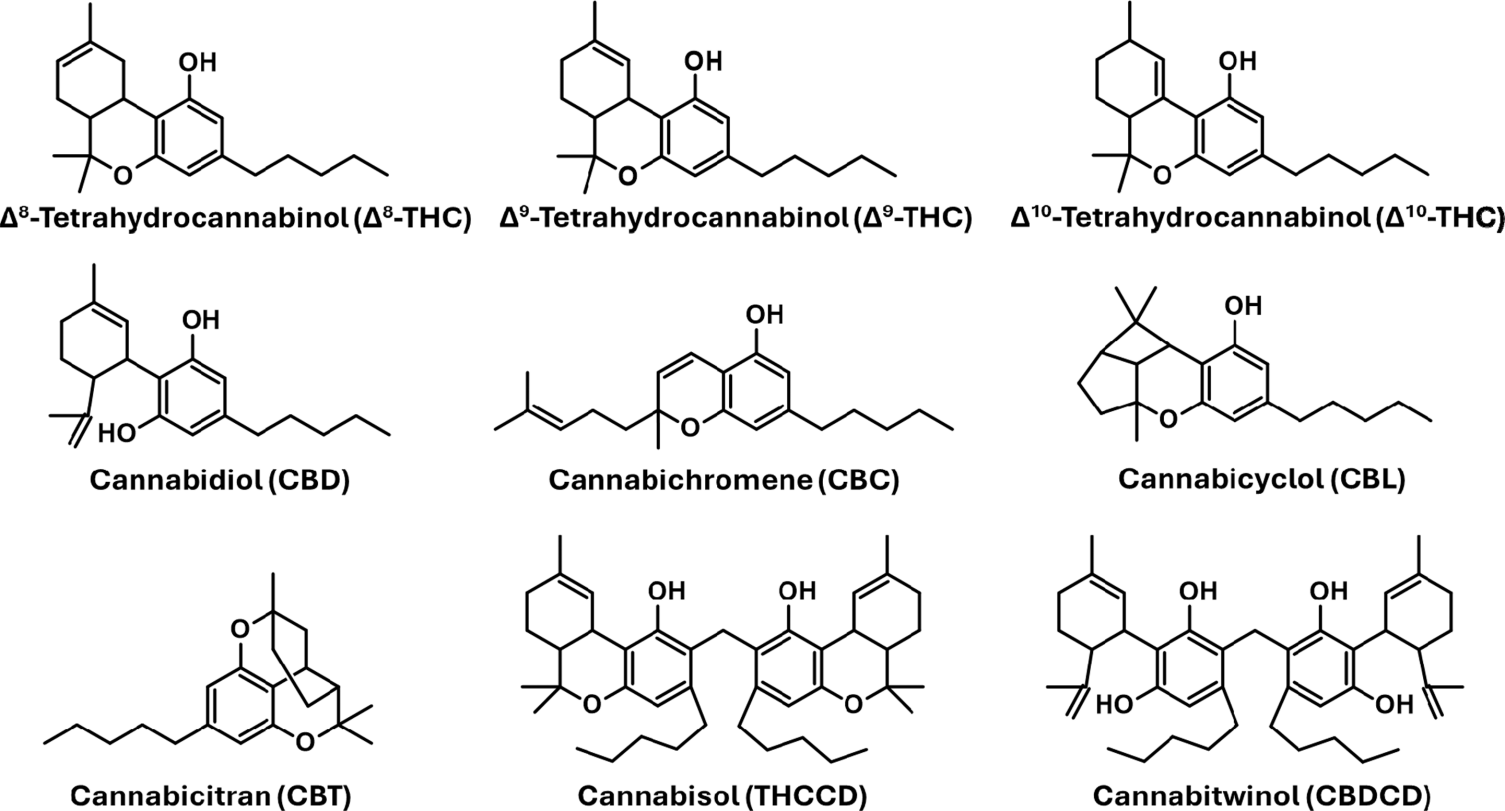
Representative cannabinoid isomers and condensed dimers of THC and CBD (THCCD and CBDCD, respectively) found in *Cannabis* plant material. The isomers share the chemical formula C_21_H_30_O_2_ (*calc*. 314.2246).

If *m*/*z* 629 is a protonated dimer of a molecule with the formula C_21_H_30_O_2_, then there are several possibilities, since this monomer formula corresponds to Δ^8^-THC, Δ^9^-THC, Δ^10^-THC, CBD, cannabichromene (CBC), cannabicyclol (CBL), and cannabicitran (CBT) (see Figure 1). These include various homodimers such as [Δ^8^-THC••Δ^8^-THC + H]^+^, [Δ^9^-THC••Δ^9^-THC + H]^+^, [CBD••CBD + H]^+^, [CBC••CBC + H]^+^, and/or [CBT••CBT + H]^+^, and various heterodimer combinations comprised of different pairs of cannabinoid isomers. On the other hand, the possibility exists for *m*/*z* 629 to represent the protonated precursor of a naturally occurring metabolite that is unique to hemp. The work presented here utilized a combination of DART-MS, computational chemistry, and field desorption MS (FD-MS) to investigate the proton-bound cannabinoid dimers formed from combinations of Δ^8^-THC, Δ^9^-THC, CBD, CBC, and CBT, and the possibility of the presence of a protonated condensed dimer. Other Δ^9^-THC isomers such as Δ^10^-THC and CBL (Figure 1) were not included, as they are present in *Cannabis* at only trace levels. The theoretical calculations provided optimized geometries, binding energies, and insights into the types of interactions that accounted for the relative stabilities of the possible complexes. The binding energy calculations and population analyses revealed the identities of the proton-bound dimers and their abundances as a function of the temperature at which the DART-MS measurements were made. Furthermore, FD-MS studies revealed that *m*/*z* 629 was not a consequence of the presence of a unique condensed dimer found only in hemp.

## 2 Experimental and Theoretical Methodology

### 2.1 Chemical Standards and Gases

Δ^8^-THC, Δ^9^-THC, CBD, CBC, and CBT cannabinoid certified reference standards in methanol were purchased from Cayman Chemical (Ann Arbor, MI, USA). Polyethylene glycol (PEG 600) was purchased from Sigma-Aldrich (St. Louis, MO, USA). Nitrogen and ultrahigh purity helium gas were obtained from AirGas (Baton Rouge, LA, USA).

### 2.2 DART-HRMS Methods

Mass spectra were collected in positive-ion mode using a DART-SVP ion source from IonSense Inc. (Saugus, MA, USA), coupled to a JEOL AccuTOF high-resolution time-of-flight (TOF) mass spectrometer (Peabody, MA, USA). The following settings were used for the DART source: grid voltage, 250 V; gas temperature, 350 °C and helium gas flow rate, 2 L/min. The following mass spectrometer settings were used: orifice 1 voltage, 20 V; ring lens voltage and orifice 2 voltage, 5 V; peak voltage, 600 V; detector voltage, 2100 V; resolving power, 10,000 full width at half-maximum (fwhm); mass spectral data collection, 2 spectra per second; and mass range, *m*/*z* 60–1000. All cannabinoid solutions were analyzed by DART-HRMS using a capillary tube sampling approach. This involved dipping the closed end of the glass melting point capillary tube (Corning Incorporated, Tewksbury, MA, USA) into the solution and presenting it to the open-air gap (i.e., the DART gas stream) between the DART ion source and mass spectrometer inlet for approximately 5 s. Each solution was analyzed in triplicate, with PEG 600 used as the mass calibrant for all acquisitions. Mass spectral translation, calibration, averaging, background subtraction, and peak centroiding were performed using msAxel software (JEOL USA, Peabody, MA). Mass spectra were analyzed using the Mass Mountaineer software suite from RBC Software (Portsmouth, NH, USA).

A 0.1 mL aliquot of Δ^8^-THC, Δ^9^-THC, CBD, CBC, and CBT certified reference materials (CRMs) (1000 μg/mL in methanol) was deposited into separate dram vials (VWR International LLC, Radnor, PA, USA). Another 0.1 mL aliquot of all five CRMs was deposited into a single dram vial, yielding a total volume of 0.5 mL of a mixture of cannabinoids. The methanol solvent in all six vials was evaporated off. The solid residue remaining in each vial (100 μg for the individual compounds and a 500 μg total for the mixture) was reconstituted in 0.05 mL (50 μL) of methanol (Sigma-Adrich, Burlington, MA). The resulting concentration for the individual Δ^8^-THC, Δ^9^-THC, CBD, CBC, and CBT compounds in solution and for the cannabinoid mixture was 2,000 μg/mL. The samples were vortexed prior to analysis by DART-HRMS to ensure full dissolution of the compounds.

### 2.3 FD-HRMS Methods

Field desorption (FD) mass spectra were acquired using a JEOL AccuTOF GC-Alpha reflectron time-of-flight mass spectrometer equipped with a combination electron ionization/field ionization/field desorption (EI/FI/FD) ion source. Approximately 1 μL of Δ^9^-THC and (separately) CBD CRMs (1000 μg/mL in methanol, Cerilliant, Round Rock, TX, USA) was applied as a liquid junction to a JEOL 10-μm field desorption emitter using a JEOL field desorption sampling device. The FD emitter was heated to 40 mA at a rate of 26 mA min^–1^ to desorb the samples. Mass spectra were acquired at a resolving power of 30,000 fwhm for the *m*/*z* range 10–800 at a spectral acquisition rate of 1 spectrum per second. The mass spectrometer was calibrated in EI mode with perfluorotripentylamine (Scientific Instrument Services, Ringoes, New Jersey, USA) for accurate mass measurements. Octadecylcyclotetrasiloxane (Sigma-Aldrich, St. Louis, MO, USA) was introduced as a mass drift correction at the last 0.5 min of the FD measurement using the automated reference inlet.

### 2.4 Computational Methods

The geometries of the starting reactants and various possible proton-bound dimer complexes were fully optimized using M06–2X hybrid density functional theory (DFT)^11^ along with the standard Pople-type 6–311++G­(2d,2p) basis set.^12,13^ It has been reported that the M06–2X functional exhibits excellent performance in determining energies and hydrogen-bonding interactions for a variety of chemical applications.^11,14–16^ The M06–2X functional is used mainly due to its high reliability and relatively low computational cost. Harmonic vibrational frequency calculations were carried out to characterize all the structures corresponding to local minima on the potential energy surface. All of the monomers and the various possible proton-bound dimer complexes examined in this study exhibited only positive frequencies, with no imaginary frequencies detected. The zero-point vibrational energy corrections employed a scaling factor of 0.967 for the M06–2X/6–311+G­(2d,2p) level.^17,18^ We also performed single-point energy calculations to get more accurate energies for the reactants and all possible proton-bound dimer complexes at the same M06–2X level using the large 6–311++G­(3df,3pd) basis set. The final energies of all the reactants and proton-bound dimers were calculated using single-point energies obtained at the M06–2X/6–311++G­(3df,3pd) level, to which a zero-point energy (ZPE) correction obtained at the M06–2X/6–311+G­(2d,2p) level was applied. The binding energies (Δ*E*_bind_) of all possible proton-bound dimer complexes were calculated using eq 1. Each proton-bound dimer binding energy was determined as the difference between the energy of the respective proton-bound dimer complex and the sum of the energies of the corresponding protonated monomer and a neutral monomer.

1 $$\Delta E_{\mathrm{bind}}=E_{\mathrm{dimer}}-\left(E_{R_{1}}+E_{R_{2}}\right)$$

In eq 1, *E*_dimer_ represents the energy of a proton-bound dimer complex, and $E_{R_{1}}$ and $E_{R_{2}}$ represent the energies of their protonated and neutral monomers, respectively. In addition, we also estimated the enthalpy (Δ*H*) for all the studied proton-bound dimer complexes using eq 2.

2 $$\Delta H=H_{\mathrm{dimer}}-\left(H_{R_{1}}+H_{R_{2}}\right)$$

In eq 2, *H*_dimer_ represents the enthalpy of a proton-bound dimer complex, and $H_{R_{1}}$ and $H_{R_{2}}$ represent the enthalpies of their protonated and neutral monomers, respectively.

The binding energies and enthalpies for all the proton-bound dimer complexes were calculated relative to their corresponding separated monomers at the M06–2X/6–311++G­(3df,3pd)//M06–2X/6–311++G­(2d,2p) level. All the electronic structure calculations were performed using Gaussian 16 software.^19^ The Gaussian output files for all the reactants, protonated monomers, and protonated dimer complexes optimized at the M06–2X/6–311++G­(2d,2p) level are provided in the Supporting Information.

We investigated the effect of temperature on the populations of the various possible proton-bound homo- and heterodimers considered in this study. The population analysis was performed by calculating the mole fractions (X_i_) of each proton-bound dimer complex using eq 3.^20,21^

3 $$X_{i}=\frac{q_{i}\exp\left(-\frac{\Delta H}{\mathrm{RT}}\right)}{\overset{n}{\underset{j=1}{\sum }}q_{j}\exp\left(-\frac{\Delta H}{\mathrm{RT}}\right)}$$

In eq 3, R is the gas constant, T is the absolute temperature (in K), and Δ*H* represents the relative heat of formation at absolute zero of the *i* ^th^ proton-bound dimer. The partition function (*q*_*i*_) is the product of the translational, vibrational, rotational, and electronic partition functions of the respective proton-bound dimer. These partition functions were calculated from the vibrational frequencies and rotational constants computed using the present M06–2X/6–311++G­(2d,2p) level calculations. Standard statistical mechanics formulas were used to calculate the partition functions.^22^ The Δ*H* values for all proton-bound dimer complexes were calculated at the M06–2X/6–311++G­(3df,3pd)//M06–2X/6–311++G­(2d,2p) level (see Table S1).

## 3 Results and Discussion

### 3.1 DART-HRMS Analysis of Hemp, Marijuana, Δ^9^-THC, and its Cannabinoid Isomers

As noted previously, hemp and marijuana can be readily and accurately differentiated through chemometric processing of their DART-HRMS profiles.^6^ Furthermore, a key mass that was found to be diagnostic for hemp specifically was nominal *m*/*z* 629. Representative DART mass spectra of hemp and marijuana, illustrating the presence and absence, respectively, of *m*/*z* 629, are shown in Figure 2. Since this mass is consistent with [2M + H^+^], where M corresponds to C_21_H_30_O_2_, and this formula in turn corresponds with that of several isomers of Δ^9^-THC, we first determined whether DART-HRMS analysis of purified reference standards of Δ^9^-THC and its isomers that are commonly found in *Cannabis* plant material would show this mass. Representative DART mass spectra of Δ^8^-THC, Δ^9^-THC, CBD, CBC, CBT, and a mixture of all five cannabinoids acquired in positive-ion mode under soft ionization conditions (i.e., an orifice 1 voltage of 20 V) are shown in Figure 2. Each of the five solutions exhibited a base peak at nominal *m*/*z* 315, consistent with the calculated protonated mass [M + H]^+^ of Δ^8^-THC, Δ^9^-THC, CBD, CBC, and CBT ([C_21_H_30_O_2_ + H]^+^) at *m*/*z* 315.2324. Lower intensity peaks in the *m*/*z* 60–314 range (i.e., lower than that of the protonated precursor ion) indicate either that some degree of fragmentation occurred or the presence of impurities. Peaks that are detected above the [M + H]^+^ (i.e., greater than *m*/*z* 315 in this case) are often indicative of the formation of various adducts. In particular, a peak at nominal *m*/*z* 629 with a relative intensity between 0.03% (in CBT, CBD, Δ^9^-THC and in the cannabinoid mixture), 0.04% in Δ^8^-THC, and 0.7% (in CBC) was detected for all samples, and its high-resolution mass was consistent with the calculated protonated mass of two precursor molecules with the chemical formula C_21_H_30_O_2_ (i.e., [(C_21_H_30_O_2_)_2_ + H]^+^ or [2M + H]^+^), corresponding to the protonated homodimers: Δ^8^-THCH^+^••Δ^8^-THC, Δ^9^-THCH^+^••Δ^9^-THC, CBDH^+^••CBD, CBCH^+^••CBC, and CBTH^+^••CBT. For the solution containing equal parts of Δ^8^-THC, Δ^9^-THC, CBD, CBC, and CBT, [2M + H]^+^ could indicate the presence of either protonated homodimers or one (or a combination) of the several possible protonated heterodimers (e.g., CBD••Δ^9^-THC, CBC••CBT, Δ^9^-THC••CBT, etc.). Given the observation that the purified forms of Δ^9^-THC and its isomers all exhibited a peak consistent with [2M + H]^+^ in their DART mass spectra, and the fact that all of these compounds are generally present in bulk plant material, we next sought to determine the structural identity of *m*/*z* 629 by assessing the relative stabilities of the possible homo- and heterodimers in order to assess which was most likely based, on their relative stabilities. This was pursued using computational methods.

**Figure 2:**
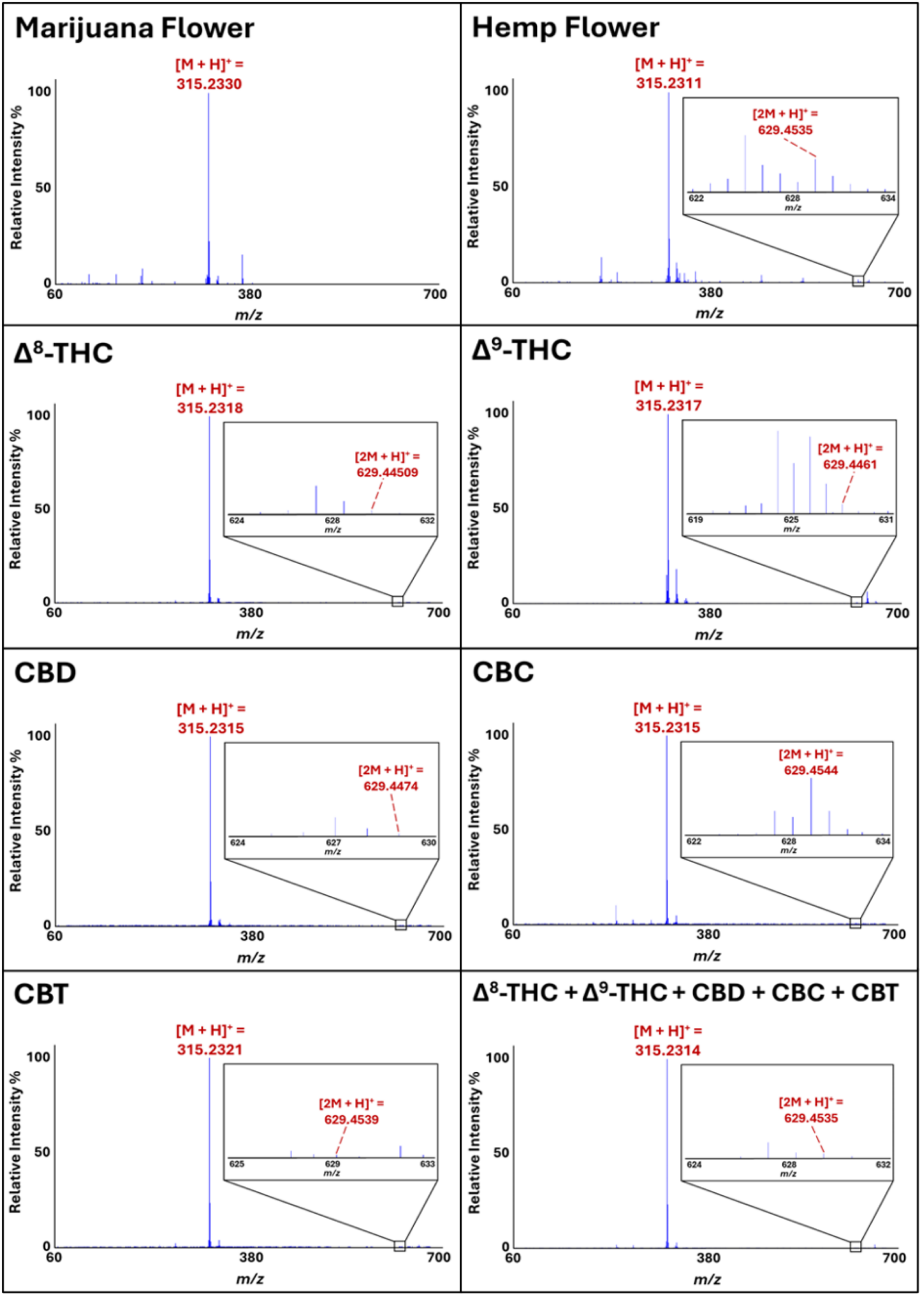
DART mass spectra of marijuana (15%w/w THC concentration) and hemp (0.07%w/w THC concentration) flowers; Δ^8^-THC, Δ^9^-THC, CBD, CBC, and CBT solutions; and a Δ^8^-THC + Δ^9^-THC + CBD + CBC + CBT mixture. Peaks at nominal *m*/*z* 315 represent [M + H]^+^ peaks, while the peaks detected at *m*/*z* 629 indicate the presence of proton-bound cannabinoid dimers [2M + H]^+^.

### 3.2 Theoretical Calculations of Cannabinoid Dimers

#### 3.2.1 Determination of the Optimized Geometries of CBD, Δ^9^-THC, CBC, Δ^8^-THC, and CBT

CBD, CBC, Δ^9^-THC, Δ^8^-THC, and CBT are constitutional isomers. Their optimized geometries, obtained at the M06–2X/6–311++G­(2d,2p) level, are shown in Figure 3. Consistent with previous studies,^23^ the structure of CBD revealed the presence of two intramolecular hydrogen bonds between the oxygen atoms of the two phenolic −OH groups and H atoms on the cyclohexene ring, with calculated O••H distances of 2.26 and 2.33 Å, respectively. In the case of Δ^9^-THC, the structure shows two intramolecular hydrogen bonds between the O atom of the phenolic −OH group and two different H atoms attached to separate carbon atoms in the cyclohexene ring, with O••H distances of 2.62 and 2.73 Å. The CBC structure has a phenolic −OH group, but the ether oxygen in its bicyclic system is not oriented or configured to enable the formation of a stable intramolecular H-bond. Consequently, CBC lacks a suitably positioned donor–acceptor pair for intramolecular hydrogen bonding. In contrast, the structure of Δ^8^-THC shows two intramolecular H-bonds between the phenolic −OH oxygen and two different H atoms on the cyclohexene ring, with O••H distances of 2.29 and 2.87 Å. The CBT structure, comprised of four fused rings, locks the molecule into a compact conformation. The two ether oxygens enhance its stability by withdrawing electron density and relieving ring strain (see Figure 3). Together, these structural features make CBT more stable than the other cannabinoids studied in this work. The relative energies computed at the ZPE-corrected M06–2X/6–311++G­(3df,3pd)//M06–2X/6–311++G­(2d,2p) level indicate that Δ^9^-THC, Δ^8^-THC, and CBT are ∼13.8, 18.1, and 19.0 kcal mol^–1^ more stable than CBD, whereas CBC is ∼0.5 kcal mol^–1^ less stable than CBD.

**Figure 3:**
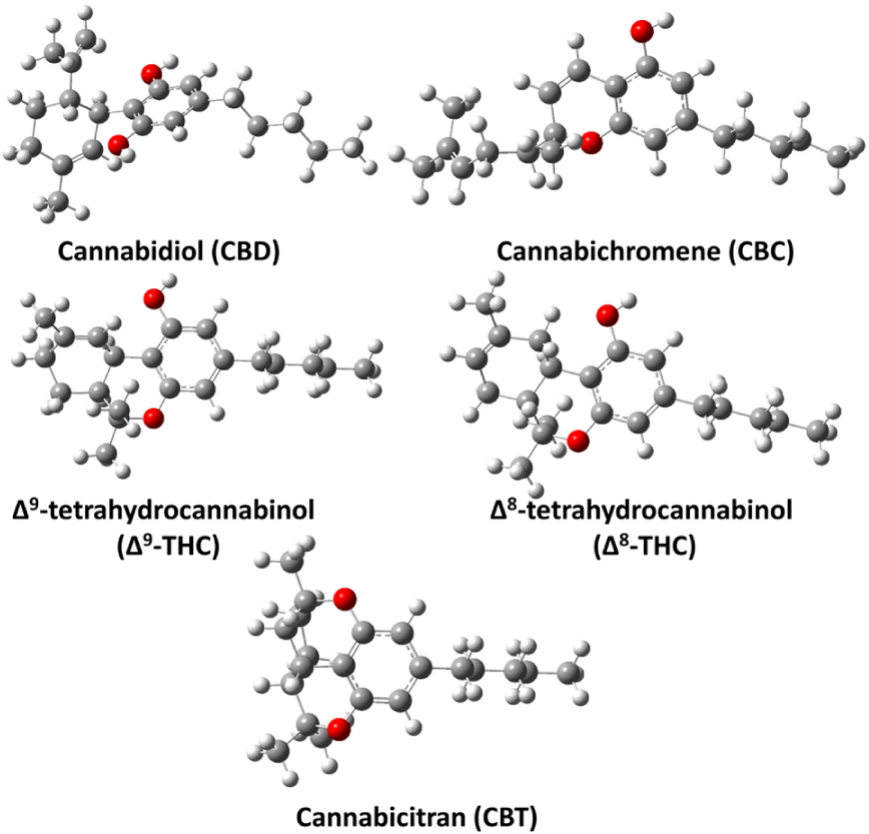
M06–2X/6–311++G­(2d,2p) level optimized geometries of CBD, CBC, Δ^9^-THC, Δ^8^-THC, and CBT. The gray, white, and red colors represent carbon, hydrogen, and oxygen atoms, respectively.

#### 3.2.2 Determination of the Optimized Geometries of Protonated CBD, Δ^9^-THC, CBC, Δ^8^-THC, and CBT

To gain deeper insight into the mass spectral characteristics observed in this study, DFT calculations were employed to investigate the potential proton-bound dimer structures of CBD, Δ^9^-THC, CBC, Δ^8^-THC, and CBT. As a preliminary step, the protonated forms of all five cannabinoid monomers were optimized to assess the structural possibilities for their corresponding dimers. Multiple protonation sites were identified in all molecules based on their distinct chemical environments and are labeled accordingly (see Figure S1). From this analysis, it was revealed that CBD and Δ^8^-THC possess 9 distinct protonation sites, while CBT, Δ^9^-THC, and CBC have 5, 10, and 11 possible protonation sites, respectively. The fully optimized structures of these protonated monomers are presented in Figures S2–S6.

#### 3.2.3 Energies and Structure Determination of Homodimers

In the next step, the structures of the possible proton-bound homodimers CBDH^+^••CBD, THCH^+^••THC, CBCH^+^••CBC, Δ^8^-THCH^+^••Δ^8^-THC, and CBTH^+^••CBT were explored at the same level of theory. The three most stable structures of each proton-bound homodimer of CBD and Δ^9^-THC, optimized at the M06–2X/6–311++G­(2d,2p) level, are shown in Figure 4, while Figure 5 presents the most stable structures of the proton-bound homodimers of CBC, Δ^8^-THC, and CBT optimized at the same level of theory. The remaining less stable structures are presented in Figures S7–S14. Using CBD in Figures 4a and S7 as a case in point, the structures of the various possible protonated CBD homodimer complexes are designated as CBDH^+^-X••CBD (X = 1–9), where X = 1–9 indicates the distinct structures as a function of the protonation sites within CBD (see Figure S1). The various possible binding interactions are shown with dashed lines between the protonated monomer of CBD and the neutral CBD molecules. Additionally, in these figures (Figures S7–S14) the binding energy values obtained at the M06–2X/6–311++G­(3df,3pd)//M06–2X/6–311++G­(2d,2p) level of theory are shown for all of the proton-bound cannabinoid homodimers.

**Figure 4:**
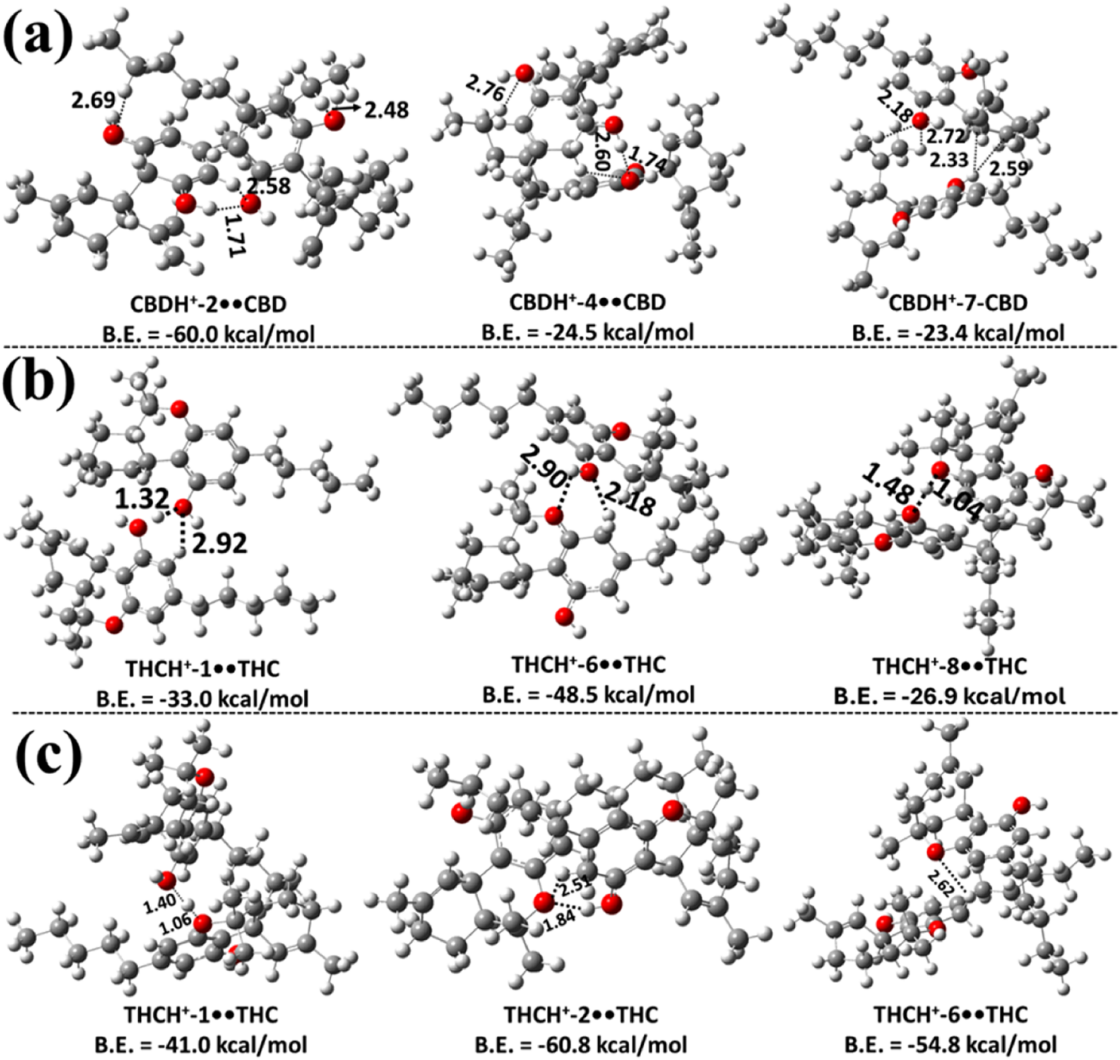
Three most stable proton-bound homodimer structures of CBD and Δ^9^-THC optimized at the M06–2X/6–311++G­(2d,2p) level of theory: (a) protonated CBD interacting with neutral CBD; (b) protonated Δ^9^-THC interacting with the −OH group of neutral Δ^9^-THC; and (c) protonated Δ^9^-THC interacting with the O atom of the pyran ring in neutral Δ^9^-THC. Bond lengths are given in Å. Binding energies (B.E.) were calculated at the M06–2X/6–311++G­(3df,3pd)//M06–2X/6–311++G­(2d,2p) level. Carbon, hydrogen, and oxygen atoms are shown in gray, white, and red, respectively.

**Figure 5:**
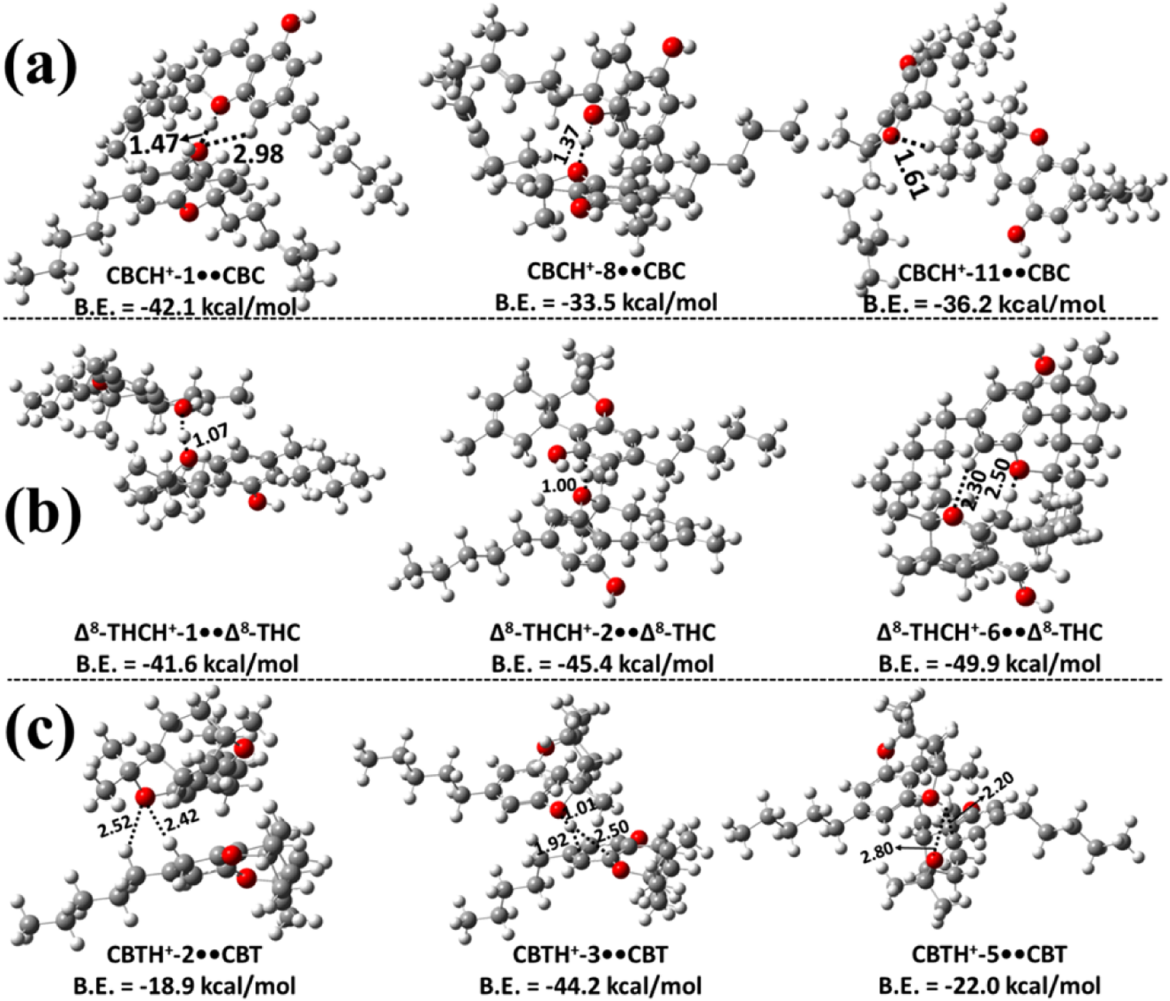
Three most stable structures of proton-bound homodimers of CBC, Δ^8^-THC, and CBT optimized at the M06–2X/6–311++G­(2d,2p) level of theory: (a) protonated CBC interacting with the O atom of the pyran ring in neutral CBC; (b) protonated Δ^8^-THC interacting with the O atom of the pyran ring in neutral Δ^8^-THC; and (c) protonated CBT interacting with neutral CBT. Bond lengths are reported in Å. Binding energies (B.E.) were calculated at the M06–2X/6–311++G­(3df,3pd)//M06–2X/6–311++G­(2d,2p) level. Carbon, hydrogen, and oxygen atoms are represented in gray, white, and red, respectively.

The results in Figure 4a and Figure S7 indicate that the structure of the CBDH^+^-2••CBD homodimer is the most stable, exhibiting a binding energy of −60.0 kcal mol^–1^. In this dimer, the protonated CBDH^+^-2 associates with the neutral CBD molecule. During the formation process, the proton that was initially bound at the C_2_ site migrates to the C_3_ site, resulting in the CBDH^+^-2••CBD dimer. The high stability of this dimer primarily arises from the intermolecular hydrogen bond between the H atom on the benzene ring of protonated CBD and the O atom of the neutral CBD, with a bond length of 2.58 Å. Additionally, three other hydrogen-bonding interactions contribute to its stability: one between the H atom of the –OH group in the protonated CBD and the O atom of the OH group in the neutral CBD molecule, with a bond length of 1.71 Å, and two other interactions between the H atom of the −CH_2_ group in the neutral CBD molecule and the O atom of the –OH group in the protonated CBD. The respective H–O bond lengths are 2.48 and 2.69 Å. Similar hydrogen-bonding interactions were observed in all other possible proton-bound homodimers of CBD identified in this work (see Figure 4a and Figure S7). The results indicate that the binding energies of these proton-bound homodimers of CBD range from 10.5 to 24.5 kcal mol^–1^ (see Figure 4a and Figure S7). This suggests that the CBDH^+^-2••CBD homodimer is ∼35.5–49.5 kcal mol^–1^ more stable than the other possible protonated CBD dimers. The calculated binding energies indicate that, except for the CBDH^+^-2••CBD species, the other less stable proton-bound homodimers are unlikely to contribute significantly to the overall population.

The proton-bound homodimers of Δ^9^-THC (THCH^+^••THC) were optimized at the same level of theory in the next step of the study. The various possible Δ^9^-THCH^+^••Δ^9^-THC structures are designated as THCH^+^-Y••THC (Y = 1–10), where Y = 1–10 corresponds to the protonation sites within THC, and they are shown in Figures 4b, 4c, S8 and S9. THC possesses a tricyclic structure that includes a benzene ring fused to a pyran ring, along with an −OH group attached to the benzene moiety. Due to this structural arrangement, proton-bound THC can engage in intermolecular interactions with a second THC molecule through two primary binding motifs. In the first type, protonated Δ^9^-THC interacts with the −OH group of the neutral Δ^9^-THC molecule via hydrogen bonding (see Figure 4b and Figure S8). In the second, the protonated Δ^9^-THC forms a hydrogen bond with the oxygen atom of the pyran ring in the neutral Δ^9^-THC molecule (see Figure 4c and Figure S9). These distinct binding configurations were explored to identify the most stable proton-bound dimer structures of Δ^9^-THC.

The results in Figures 4b and S8 indicate that THCH^+^-6••THC is the most stable homodimer, with a binding energy of −48.5 kcal mol^–1^, compared to the other possible homodimers of Δ^9^-THC. In this dimer, the protonated THCH^+^ interacts with the −OH group of the neutral THC. During geometry optimization, it was observed that the proton originally located at the C_6_-position shifted to the C_5_-position, leading to the formation of the THCH^+^-6••THC dimer (see Figure S1 and Figure 4b). The optimized structure reveals an intermolecular ionic hydrogen bond (IHB) between the H atom of THCH^+^ and the O atom of the neutral THC (2.18 Å), as well as an additional hydrogen-bonding interaction between the O atom of the pyran ring in the neutral THC and the H atom of the −OH group of the protonated THC, with a bond length of 2.90 Å. We observed similar IHBs and normal hydrogen-bonding interactions in all of the other possible homodimers of the protonated THC, and in its interactions with the −OH group of the neutral THC molecule. Based on the results, the THCH^+^-6••THC dimer was found to be more stable than the others by 15.5–35.4 kcal mol^–1^.

From the results shown in Figures 4c and S9, the binding energy of the THCH^+^-2••THC dimer was −60.8 kcal mol^–1^, indicating greater stability compared to the other possible homodimers, where the protonated THC interacts with the O atom of the pyran ring in the neutral THC molecule. During the geometry optimization, it was observed that the proton initially located at the C_2_ position migrated to the C_3_ position, resulting in the formation of the THCH^+^-2••THC dimer (see Figures 4c and S3). The optimized structure reveals the presence of an IHB between the protonated Δ^9^-THC hydrogen and the oxygen atom of the pyran ring in the neutral Δ^9^-THC, with a bond length of 2.51 Å. Additionally, a conventional hydrogen bond is formed between the pyran ring O atom of the neutral Δ^9^-THC and the −OH hydrogen of the protonated Δ^9^-THC, with a bond length of 1.84 Å. These interactions make the THCH^+^-2••THC dimer more stable by ∼6.0–46.5 kcal mol^–1^ compared to the other dimer configurations. Similar interaction patterns were identified in other potential homodimers formed from Δ^9^-THC. Binding energy calculations suggest that dimers involving protonated Δ^9^-THC interacting with the pyran ring O atom of neutral Δ^9^-THC are energetically more favorable than those involving interactions with the −OH group of the neutral Δ^9^-THC molecule.

Various possible proton-bound homodimers of CBCH^+^••CBC formed from a protonated CBC and a neutral CBC molecule were studied, and the corresponding protonated CBC monomer structures are shown in Figure S4. The optimized structures of the various possible proton-bound homodimers formed through the interaction of protonated CBC with the O site or the −OH site of the CBC monomer are shown in Figure 5a, and Figures S10 and S11, respectively. In these figures, the structures of various possible CBCH^+^••CBC homodimers, labeled as CBCH^+^-Z••CBC (Z = 1–11), where Z corresponds to the protonation sites within CBC, are shown in Figures S1 and S4. From Figure 5a, the most stable structure was found to be CBCH^+^-1••CBC, with a binding energy of −42.1 kcal mol^–1^. This stability is primarily due to a strong intermolecular IHB formed between the hydrogen atom of the protonated oxygen in the phenolic group and the oxygen atom of the dihydropyran ring in the neutral CBC, with a bond length of 1.47 Å. In addition, there is another intermolecular hydrogen-bonding interaction between the O atom of a hydroxyl group in the protonated CBC and an H atom on the phenolic ring of the neutral CBC, with a bond length of 2.98 Å. The results reveal that CBCH^+^-1••CBC is ∼16.0–28.6 kcal mol^–1^ more stable compared to the values of other possible dimers in this class. The stability of other possible protonated dimer complexes is primarily a consequence of intermolecular IHB interactions that resemble those present in the structure of CBCH^+^-1••CBC (see Figure 5a and Figure S10).

Several potential CBCH^+^••CBC dimers, formed by the interaction of protonated CBC with the −OH group of CBC, were also explored. According to the results presented in Figure S11, the CBCH^+^-1••CBC structure is the most stable among the dimers in this series, with a binding energy of about −32.9 kcal mol^–1^ relative to that of the initial reactants. This stability arises from the presence of an intermolecular IHB between the protonated H atom of the hydroxyl group in CBC and the O atom in the hydroxyl group of another CBC. The binding energies of the other remaining protonated dimers of CBC were found to range between −10.4 and −25.4 kcal mol^–1^. These values indicate that these complexes are ∼7.5–22.5 kcal mol^–1^ less stable than the CBCH^+^-1••CBC structure. Similar ionic and normal hydrogen bonding interactions were observed in all protonated CBC dimer complexes (see Figure S11).

Various possible proton-bound homodimers of Δ^8^-THC were formed via the interaction of protonated Δ^8^-THC with the O site or the −OH site of another Δ^8^-THC. The various possible proton-bound homodimers of Δ^8^-THCH^+^-N••Δ^8^-THC (N = 1–9) that were obtained are shown in Figures 5b, S12 and S13. Figures 5b and S12 indicate that Δ^8^-THCH^+^-6••Δ^8^-THC is the most stable, with a binding energy of −49.9 kcal mol^–1^. The structure of this dimer indicates a strong IHB interaction between Δ^8^-THCH^+^-6 and the O site of another Δ^8^-THC molecule, with a corresponding bond length of 2.50 Å (see Figure 5b). Additionally, hydrogen bonding interactions were observed with a bond length of 2.30 Å. This dimer structure was found to be more stable than the other possible dimers by ∼4.5–31.3 kcal mol^–1^. The less stable homodimers of this class were found to have similar types of interactions (see Figure 5b and Figure S12). Figure S13 illustrates various proton-bound homodimers of Δ^8^-THCH^+^••Δ^8^-THC, which are formed via the interaction of protonated Δ^8^-THCH^+^ with the OH moiety of neutral Δ^8^-THC. The most stable homodimer structure was identified as a complex formed between Δ^8^-THCH^+^-1 and the −OH group of another Δ^8^-THC (Figure S13), with a binding energy of about −33.5 kcal mol^–1^. The binding energies of other possible dimer configurations ranged from −6.6 to −30.0 kcal mol^–1^. These results suggest that dimers formed via the interaction of protonated Δ^8^-THC with the O site of another Δ^8^-THC monomer are generally more stable than those formed through interaction with the −OH site (see Figure 5b, S12, and S13).

The proton-bound homodimers of CBTH^+^-M••CBT (M = 1–5) were explored by considering all possible protonated CBT isomers (see Figure S6) in combination with neutral CBT. Multiple proton-bound homodimer structures were identified, with the three most stable configurations presented in Figure 5c, and the remaining ones shown in Figure S14. Among these, the CBTH^+^-3••CBT complex was found to be the most stable, exhibiting a binding energy of ∼–44.2 kcal mol^–1^, which is significantly higher than that of the other candidate dimers. Structural analysis revealed that the proton from CBTH^+^-3 is transferred to the O atom of the pyran ring of the adjacent CBT molecule, forming a short O–H bond of 1.01 Å. This proton simultaneously engages in stabilizing interactions with the neighboring benzene ring carbon atoms, enhancing the overall binding strength. Energetically, CBTH^+^-3••CBT is more stable by ∼22.2–31.2 kcal mol^–1^ than the other homodimers of CBT investigated in this study (see Figure 5c and S14).

#### 3.2.4 Energies and Structure Determination of Heterodimers

We investigated the various proton-bound heterodimers that CBD, Δ^9^-THC, CBC, Δ^8^-THC, and CBT can form with one another. We first examined the proton-bound heterodimers of CBD and Δ^9^-THC (CBDH^+^••Δ^9^-THC and Δ^9^-THCH^+^••CBD) using the same aforementioned computational approach. Structurally, CBD features a benzene ring connected to a cyclohexene ring, with two −OH groups attached to the benzene moiety, while THC possesses a fused tricyclic ring system in which a benzene ring is fused to a pyran ring and contains a single −OH group. These structural differences result in distinct interaction modes when forming heterodimers. Protonated CBD can interact with Δ^9^-THC in two primary ways: either through hydrogen bonding interactions with the −OH group or via interaction with the O atom of the pyran ring in Δ^9^-THC. Similarly, protonated Δ^9^-THC can also form heterodimers with neutral CBD molecules. The most stable proton-bound heterodimers of the CBDH^+^-X••Δ^9^-THC (X = 1–9) and Δ^9^-THCH^+^-Y••CBD (Y = 1–10) types were optimized at the M06–2*X*/6–311++G­(2d,2p) level of theory and are presented in Figure 6. Additional less stable heterodimer structures are shown in Figures S15 - S17. The results indicate that CBDH^+^-3••Δ^9^-THC is the most stable dimer among those investigated in this class, with a binding energy of −51.8 kcal mol^–1^. The stability of this dimer is primarily due to the presence of an intermolecular IHB between the protonated hydrogen atom of the protonated CBD and the OH group oxygen atom of Δ^9^-THC, with a bond length of 2.30 Å. Three other hydrogen bonds are also observed in this complex: one between the OH group oxygen atom of the protonated CBD and two different hydrogen atoms of the cyclohexene ring in THC, with bond lengths of 2.60 and 2.87 Å, respectively; and another between the O atom of the pyran ring and the H atom of the −CH_2_ group in protonated CBD, with a bond length of 2.91 Å (see Figure 6a). The binding energies of other possible protonated dimers in this class, formed through the interaction of protonated CBD with the −OH moiety of Δ^9^-THC, range between −10.3 and −26.8 kcal mol^–1^ (see Figure 6a and Figure S15). All of these protonated dimer complexes exhibited IHB and normal hydrogen bonding interactions to achieve stability. These binding energies indicate that they are less stable than CBDH^+^-3••THC. Hence, they would not significantly contribute to the population.

**Figure 6:**
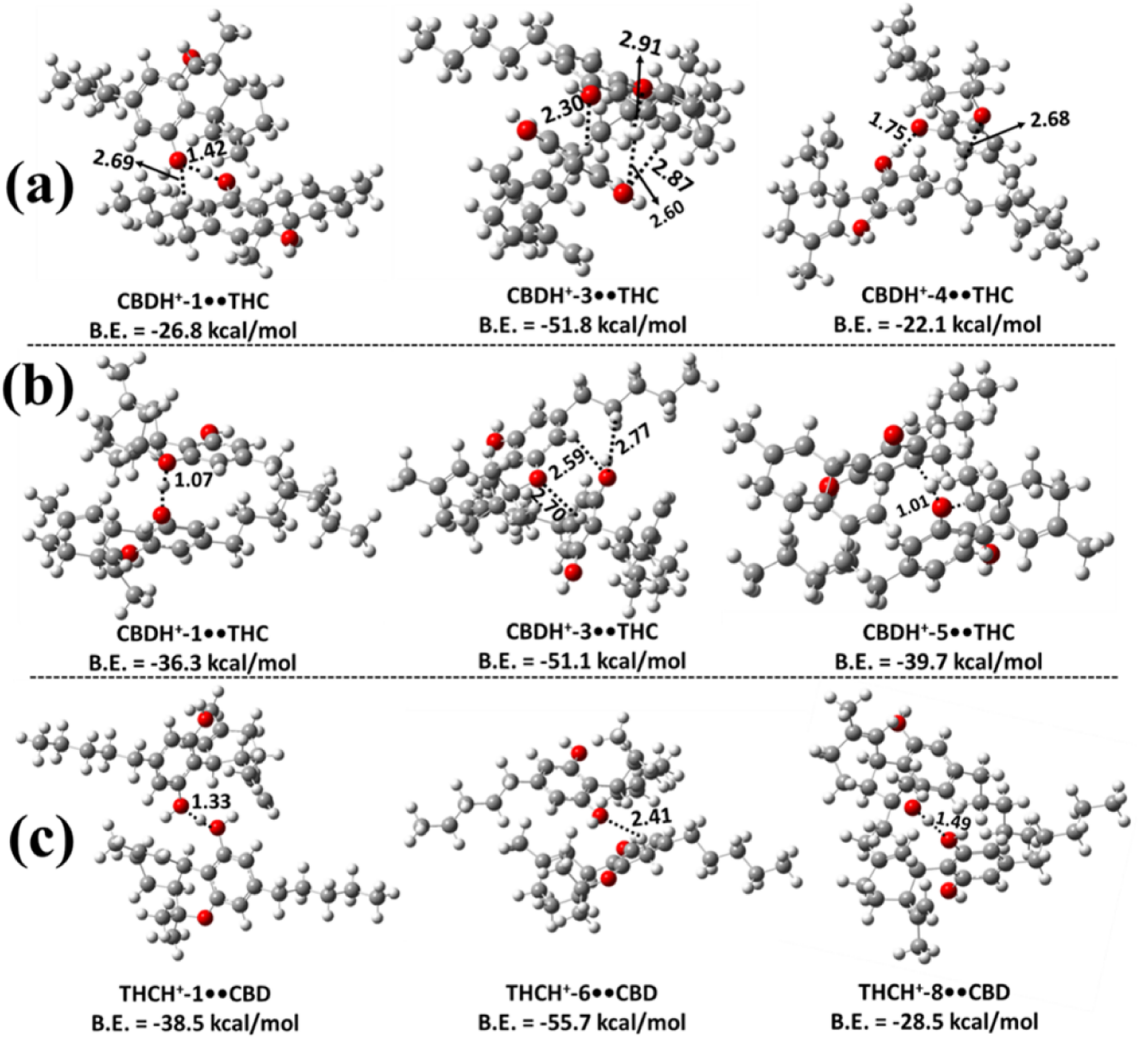
Three most stable proton-bound heterodimer structures of CBD and Δ^9^-THC optimized at the M06–2X/6–311++G­(2d,2p) level of theory: (a) protonated CBD interacting with the −OH moiety of neutral Δ^9^-THC; (b) protonated CBD interacting with the O atom of the pyran ring in neutral Δ^9^-THC; and (c) protonated Δ^9^-THC interacting with neutral CBD. Bond lengths are reported in Å. Binding energies (B.E.) were calculated at the M06–2X/6–311++G­(3df,3pd)//M06–2X/6–311++G­(2d,2p) level. Carbon, hydrogen, and oxygen atoms are represented in gray, white, and red, respectively.

Based on the results shown in Figures 6b and S16, the CBDH^+^-3••THC was found to be more stable, with a binding energy of −51.1 kcal mol^–1^ compared to other possible dimers obtained when the protonated CBD interacts with the O atom of the pyran ring in a neutral Δ^9^-THC. Formation of intermolecular IHB interactions was found between the protonated H atom of the protonated CBD and the hydroxy O atom of the neutral Δ^9^-THC molecule, with a bond length of 2.70 Å. Additionally, in the CBDH^+^-3••THC dimer complex, we observed intermolecular hydrogen bonds with O–H bond lengths of 2.59 and 2.77 Å. The binding energies of the other possible dimers in this category were found to range between −14.3 and −39.7 kcal mol^–1^, which indicates that they are ∼14.8 – 37.0 kcal mol^–1^ less stable than the CBDH^+^-3••THC dimer complex. The present calculated energies indicate that the heterodimers formed from the interaction of protonated CBD with the O atom of the pyran ring of Δ^9^-THC were more stable than the heterodimers formed through the interaction of protonated CBD with the −OH moiety of Δ^9^-THC (see Figures 6a, 6b, S15 and S16).

The three most stable heterodimer structures formed when protonated Δ^9^-THC interacts with the neutral CBD molecule are shown in Figure 6c. Among them, the THCH^+^-6••CBD dimer is the most stable, exhibiting a binding energy of −55.7 kcal mol^–1^. The structure clearly reveals the formation of an intermolecular IHB between the protonated hydrogen atom of Δ^9^-THC and the hydroxyl O atom of the neutral CBD, with a bond length of 2.41 Å. Similar ionic and conventional hydrogen bonding interactions were observed across all THCH^+^••CBD dimers. Based on binding energy comparisons, THCH^+^-6••CBD was found to be ∼17.1–45.3 kcal mol^–1^ more stable than the other THCH^+^••CBD dimers. This suggests that the less stable heterodimers in this class (see Figure S17) are unlikely to contribute significantly to the overall population under equilibrium conditions. We also found that the binding energy of the THCH^+^-6••CBD complex is more stable by 3.9–4.6 kcal mol^–1^ than the stable CBDH^+^-3••THC dimers (see Figure 6).

We then examined the proton-bound heterodimers of CBD and Δ^9^-THC with CBC, Δ^8^-THC, and CBT. Computational results indicate that CBDH^+^-2 and Δ^9^-THCH^+^-2 preferentially form strong complexes with CBC, Δ^8^-THC, and CBT. The most stable, fully optimized structures of these dimers are depicted in Figure 7. Specifically, Figure 7a illustrates the most stable CBD-based heterodimers, in which CBDH^+^-2 interacts with CBC, Δ^8^-THC, or CBT monomers. Figure 7b shows the analogous Δ^9^-THC-based dimers, formed by the association of Δ^9^-THCH^+^-3 with CBC, Δ^8^-THC, or CBT monomers. Only the most stable species within each set were analyzed further because the less stable isomers were predicted to have negligible populations. Among all candidates, CBDH^+^-2••CBC and Δ^9^-THCH^+^-3••Δ^8^-THC were the most stable, with binding energies of −58.6 and −45.4 kcal mol^–1^, respectively. These are ∼2.9–35.2 kcal mol^–1^ and 3.8–12.8 kcal mol^–1^ more stable than the other possible heterodimers in their respective series. Structural analysis suggests that their enhanced stability arises from a combination of hydrogen bonding and strong interactions between the proton and the π-electron density of the benzene ring (see Figure 7).

**Figure 7:**
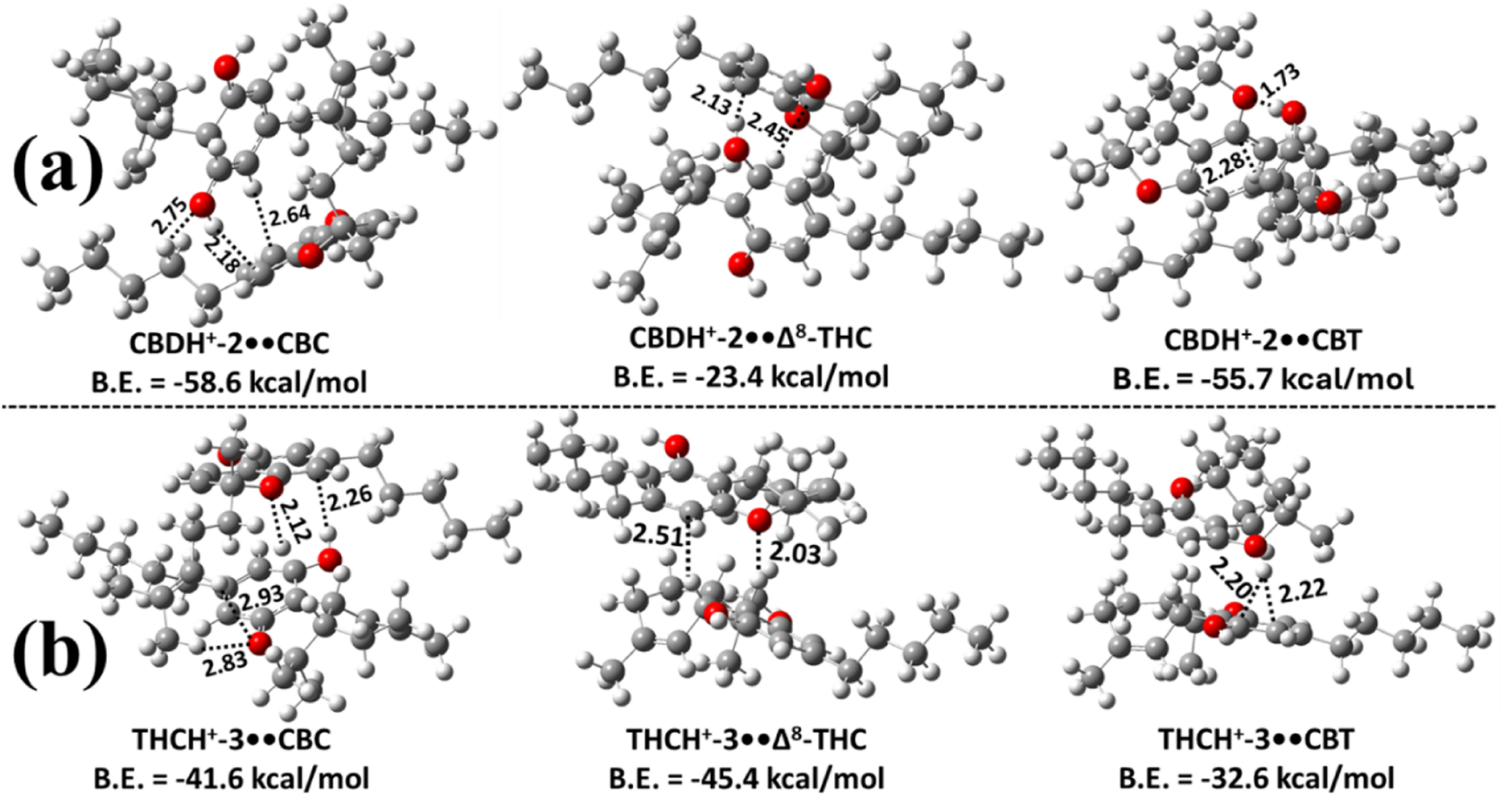
Most stable structures of the proton-bound heterodimers of CBD and Δ^9^-THC with CBC, Δ^8^-THC, and CBT optimized at the M06–2X/6–311++G­(2d,2p) level: (a) protonated CBD interacting with the O atom of the pyran ring in neutral CBC, Δ^8^-THC, and CBT, respectively; (b) protonated Δ^9^-THC interacting with the O atom of the pyran ring in neutral CBC, Δ^8^-THC, and CBT, respectively. Bond lengths are reported in Å. Binding energies (B.E.) were calculated at the M06–2X/6–311++G­(3df,3pd)//M06–2X/6–311++G­(2d,2p) level. Carbon, hydrogen, and oxygen atoms are shown in gray, white, and red, respectively.

Next, we explored the potential formation of proton-bound heterodimers of CBC with CBD, Δ^9^-THC, Δ^8^-THC, and CBT. All possible proton-bound heterodimers were optimized by considering protonated CBC monomers interacting with any one of the neutral molecules (CBD, Δ^9^-THC, Δ^8^-THC, or CBT) at the same level of theory. The most stable proton-bound heterodimers, namely CBCH^+^••CBD, CBCH^+^••Δ^9^-THC, CBCH^+^••Δ^8^-THC, and CBCH^+^••CBT, are presented in Figure 8a. The less stable proton-bound dimers were not considered further due to their expected negligible population contributions. From Figure 8a, CBCH^+^-1••CBD was identified as the most stable complex, with a binding energy of −42.8 kcal mol^–1^, which is ∼1–2 kcal mol^–1^ more stable than the other most stable proton-bound heterodimers of CBC. Additionally, all structures indicate that CBCH^+^-1 forms the most stable complexes with the other cannabinoids (CBD, Δ^9^-THC, Δ^8^-THC, and CBT). The interactions in these heterodimers are characterized by strong IHBs, with bond lengths ranging from 1.04 to 1.47 Å (see Figure 8a).

**Figure 8:**
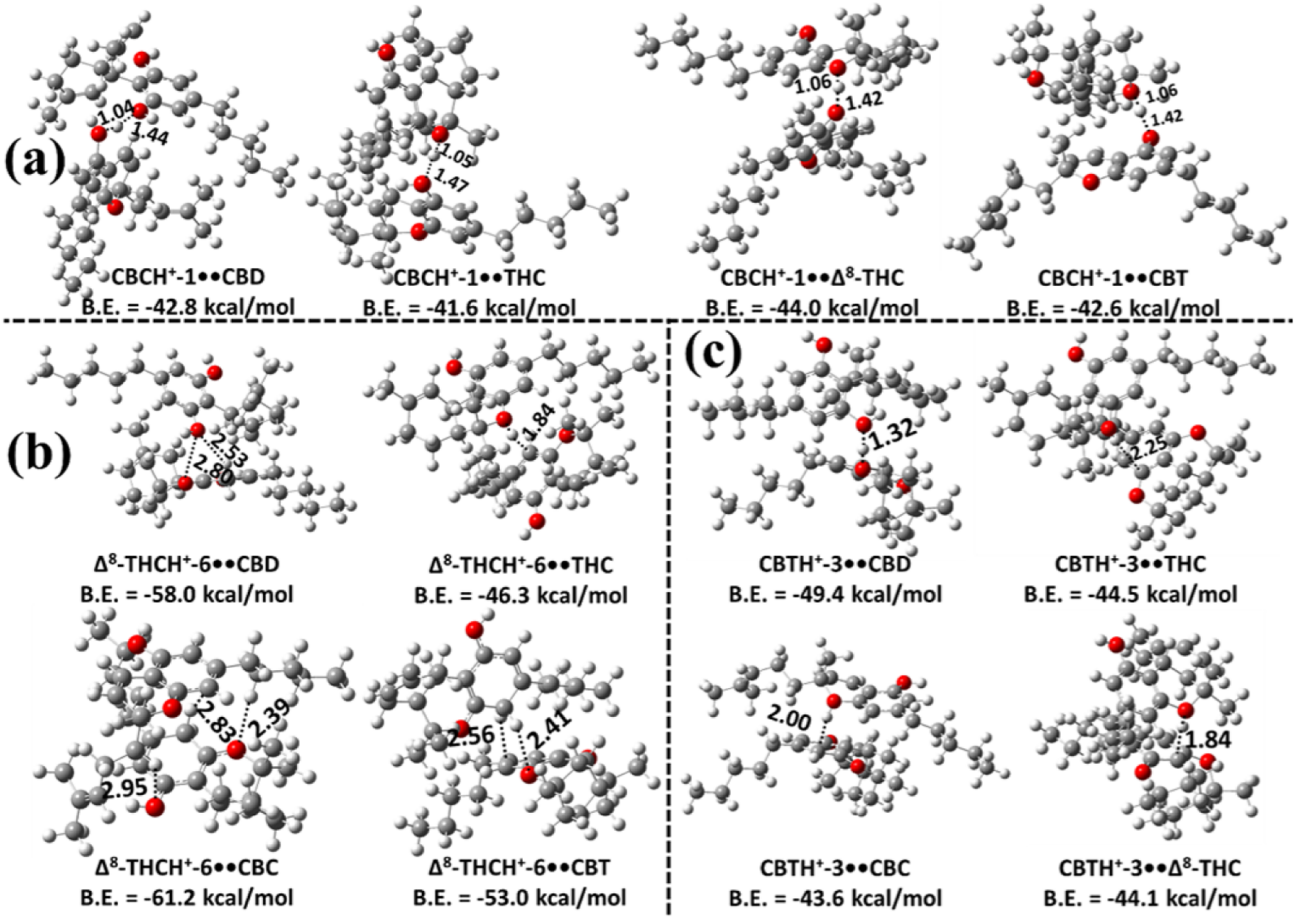
Most stable structures of the proton-bound heterodimers of CBC, Δ^8^-THC, and CBT optimized at the M06–2X/6–311++G­(2d,2p) level: (a) most stable structures of the proton-bound heterodimers of CBC with CBD, Δ^9^-THC, Δ^8^-THC, and CBT; (b) most stable structures of the proton-bound heterodimers of Δ^8^-THC with CBD, Δ^9^-THC, CBC, and CBT; and (c) most stable structures of the proton-bound heterodimers of CBT with CBD, Δ^9^-THC, CBC, and Δ^8^-THC. Bond lengths are reported in Å. Binding energies (B.E.) were calculated at the M06–2X/6–311++G­(3df,3pd)//M06–2X/6–311++G­(2d,2p) level. Carbon, hydrogen, and oxygen atoms are represented in gray, white, and red, respectively.

We investigated the heterodimer formation of proton-bound Δ^8^-THC with CBD, Δ^9^-THC, CBC, and CBT. All possible dimers were optimized using the same computational protocol, considering protonated Δ^8^-THC species paired with neutral CBD, Δ^9^-THC, CBC, or CBT molecules. The most stable configurations of Δ^8^-THCH^+^••CBD, Δ^8^-THCH^+^••Δ^9^-THC, Δ^8^-THCH^+^••CBC, and Δ^8^-THCH^+^••CBT are shown in Figure 8b, while the less stable dimers were excluded due to their negligible contributions to the dimer population (because of their low binding energies). Among these, the Δ^8^-THCH^+^-6••CBC complex is the most stable, with a binding energy of –61.2 kcal mol^–1^, which is ∼3.2–14.9 kcal mol^–1^ more stable than the other proton-bound Δ^8^-THC dimers. In these complexes, the proton on Δ^8^-THCH^+^-6 frequently shifts either to an adjacent benzene carbon or to the pyran-ring oxygen of the Δ^9^-THC or CBT molecules, forming strong IHBs (see Figure 8b). Additional interactions in these complexes include conventional hydrogen bonding and proton-π interactions with the benzene ring of the neutral partner (see Figure 8b).

The formation of proton-bound heterodimers between protonated CBT (CBTH^+^) and the neutral cannabinoids CBD, Δ^9^-THC, CBC, and Δ^8^-THC was investigated in the next step. The most stable complexes such as CBTH^+^••CBD, CBTH^+^••Δ^9^-THC, CBTH^+^••CBC, and CBTH^+^••Δ^8^-THC are presented in Figure 8c. Among the protonation sites of CBT, the CBTH^+^-3 isomer exhibited the strongest binding with all four cannabinoids. The CBTH^+^-3••CBD heterodimer was found to be the most stable, with a binding energy of −49.4 kcal mol^–1^, which is ∼4.9–5.8 kcal mol^–1^ lower than the other heterodimers. Structural analysis revealed that the proton transfers from the ring carbon atom of CBTH^+^-3 to the oxygen atom of the hydroxyl or pyran ring in the neutral partner. This proton then forms a strong intermolecular interaction with the O atoms and also interacts with the π-electron density of the benzene ring in the neutral molecule, with bond distances ranging from 1.32 to 2.25 Å.

#### 3.2.5 Computational Results Summary

The present results suggest that any of the protonated monomers can interact with CBD, Δ^9^-THC, CBC, Δ^8^-THC, or CBT in multiple orientations. The calculated binding energies indicate that proton-bound dimers formed from the interactions at the O site of the pyran ring in Δ^9^-THC, CBC, Δ^8^-THC, and CBT are the most stable. The results also indicate that CBDH^+^-2••CBD and Δ^9^-THCH^+^-2••Δ^9^-THC, with binding energies of −60.0 and −60.8 kcal mol^–1^, respectively, are more stable than all other possible homodimers studied. In contrast, Δ^8^-THCH^+^-6••CBC, with a binding energy of −61.2 kcal mol^–1^, is more stable than all of the other heterodimers examined here. Furthermore, the results indicate that Δ^8^-THCH^+^-6••CBC is ∼ 0.4 – 1.2 kcal mol^–1^ more stable than the most stable homodimers, such as CBDH^+^-2••CBD and Δ^9^-THCH^+^-2••Δ^9^-THC found in this study.

### 3.3 Population Analysis

An important factor to consider in investigating the structural identity of *m*/*z* 629 is the potential influence of temperature on the stability, and by extension, the population, of the most stable protonated dimers observed in this work, because the observation of this mass by DART-HRMS occurred at a temperature setting of 350 °C. Thus, we examined how temperature influences the populations of the various proton-bound homo- and heterodimers formed by CBD, Δ^9^-THC, CBC, Δ^8^-THC, and CBT. The population distribution was determined by computing the mole fractions (X_i_) of each proton-bound dimer complex using eq 3. The resulting mole fraction values (in percent) for all complexes were then plotted over the 50–800 K temperature range, as illustrated in Figure 9. The results indicate that the relative concentration of the most stable Δ^8^-THCH^+^-6••CBC (red line) dimer decreases markedly with increasing temperature, from 98.8% at 50 K to 4.5% at 800 K. The second most stable complex, Δ^9^-THCH^+^-2••Δ^9^-THC (pink line), shows a slight increase in population from 1% at 50 K to 2% at 100 K; however, its mole fraction becomes negligible at higher temperatures. The third most stable complex, CBDH^+^-2••CBD (navy line), exhibits the opposite trend. Its population is negligible between 50 and 100 K but increases significantly with temperature. For instance, its mole fraction increases from 3% at 100 K to 78% at 500 K, before decreasing to 55% at 800 K (see Figure 9).

**Figure 9:**
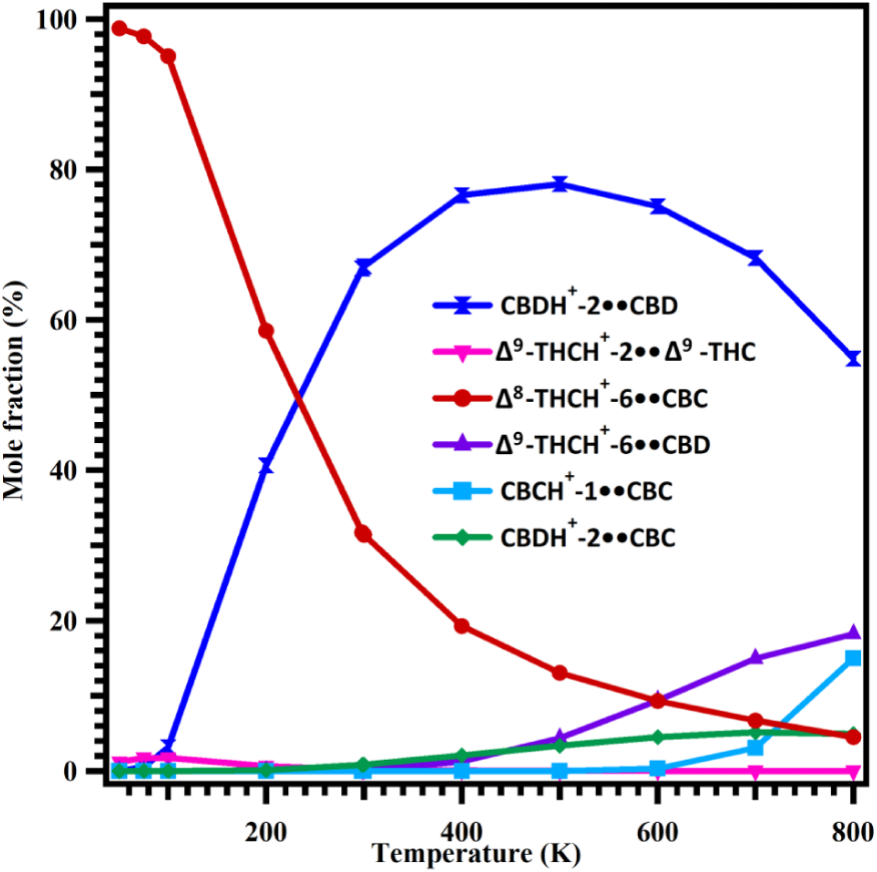
Relative concentrations of the major proton-bound homo- and heterodimers of CBD, Δ^9^-THC, CBC, Δ^8^-THC, and CBT over the temperature range of 50–800 K.

Even though the binding energy differences among these three complexes are small (see Figures 8b and 4), their populations differ significantly across the studied temperature range. The high population of CBDH^+^-2••CBD arises primarily from the substantially larger contributions of its vibrational partition functions compared to those of Δ^8^-THCH^+^-6••CBC and Δ^9^-THCH^+^-2••Δ^9^-THC over the same temperatures. The results also indicate that the mole fractions of Δ^9^-THCH^+^-6••CBD (purple line) and CBDH^+^-2••CBC (green line) are negligible in the 50–400 K range (see Figure 9). Beyond 400 K, the concentrations of Δ^9^-THCH^+^-6••CBD (purple line) and CBDH^+^-2••CBC (green line) increase from 1% to 18% and from 2% to 5%, respectively, over the 400–800 K range. The mole fraction of CBCH^+^-1••CBC (teal line) remains negligible between 50 and 600 K but rises from 3% to 15% between 600 and 800 K. Overall, the results indicate the following: (1) the most stable Δ^8^-THCH^+^-6••CBC is the dominant species below 240 K; and (2) CBDH^+^-2••CBD becomes the most abundant dimer in the 240–800 K range. However, an important consideration in this regard is the previously reported observation that the temperature of the DART gas stream (between the ion source and mass spectrometer inlet where samples are introduced) varies from that set by the analyst, in that it is often lower depending on where the sample is introduced.^24^ For the instrument employed in this study, it was determined that the temperature of the metastable helium gas stream exiting the DART and measured between the ion source and the mass spectrometer inlet (see Figure S18) is approximately 100 °C lower than the 350 °C set for the DART hardware. This raises the question of whether the falloff in temperature that occurs in the sampling gap would result in a change in the identity of the most abundant dimer. However, as the results in Figure 9 show, the CBD dimer is dominant over a temperature range that is broad enough to include DART gas stream temperatures that are lower by more than 300 °C than the 350 °C set by the analyst. This is an important observation that reveals that, in this instance, the DART gas temperature variability observed in the sampling space between the ion source and MS inlet does not change the results.

We noted earlier that the DART-HRMS analyses were conducted in triplicate and that, in all spectra, *m*/*z* 629 was consistently observed. However, because the mole fraction results reported in Figure 9 were computationally derived, the consistency of the relative intensity trend for this mass is not reflected through the presence of error bars. The enthalpies (ΔH) for all proton-bound dimer complexes were computed at the M06–2X/6–311++G­(3df,3pd)//M06–2X/6–311++G­(2d,2p) level, and in this case, the primary source of error would arise from the calculated ΔH values. The use of the M06–2X functional with the large 6–311++G­(3df,3pd) basis set is expected to introduce an uncertainty of ∼1–2 kcal mol^–1^ in Δ*H* values.^25^ To assess the effect of this uncertainty on the population distribution, we adopted an upper bound value of ±2 kcal mol^–1^ and performed a sensitivity analysis by systematically increasing and decreasing the ΔH values of all proton-bound dimer complexes by 2 kcal mol^–1^. The results show that uniformly increasing or decreasing the ΔH values of all proton-bound dimers by 2 kcal mol^–1^ does not alter their relative mole fractions as calculated using eq 3. This is because the same systematic error is applied to all proton-bound dimer complexes, and consequently, their mole fractions are unaffected. Therefore, inclusion of error bars in Figure 9 is not appropriate, as the assumed theoretical uncertainty does not lead to any meaningful variation in the calculated population distribution.

### 3.4 FD-MS Analysis of Hemp and Marijuana Samples

Another possible identity of the *m*/*z* 629 marker for hemp that was observed by DART-HRMS analysis, is that it may be a hemp-specific biomarker. If this is the case, it would not be expected to be detected via DART-HRMS analysis of purified authentic standards of Δ^9^-THC, CBD, and their various isomers. However, we did detect this mass in all of the certified reference standards that we analyzed by DART-HRMS, despite the fact that they were generated by synthesis (as opposed to extraction from plant sources that might have been contaminated with trace levels of a putative *m*/*z* 629 biomarker). This argues against the mass being a hemp-specific natural product. Nevertheless, we explored the possibility of the presence of a hemp-derived *m*/*z* 629 biomarker by FD-MS analysis of CBD and Δ^9^-THC reference standards derived from synthesis, as well as hemp and marijuana plant materials. FD^26,27^ is a sample introduction and ionization method first reported by Beckey in 1969 ^28^ as a refinement to field ionization (FI).^29–31^ Field ionization relies on electron tunneling from gas-phase molecules to an emitter constructed of a wire with carbon nanowhiskers. Ionization occurs when a high voltage is applied to the emitter, resulting in very high electric fields at the sharp tips of the nanowhiskers.^32^ FI is a soft ionization method that produces molecular ions from organic molecules with little or no fragmentation, and field desorption refers to the case where samples are applied directly onto the emitter. Importantly, FD can produce molecular ions and nonprotonated dimers from large saturated or unsaturated hydrocarbons. Therefore, the method was deemed suitable for probing the presence of an *m*/*z* 629 biomarker because: (a) if such a molecule was present in the plant material, it would be detected as a molecular ion (cation radical) at nominal *m*/*z* 628; and (b) if *m*/*z* 629 was instead a gas-phase dimer generated during the DART-MS measurement, then it would be detected at nominal *m*/*z* 628 in the FD analysis of not only the hemp and marijuana plant material but also in the purified reference standards derived by synthesis, which should not contain this putative natural product.

Figure S19 shows the FD mass spectra of authentic standards of CBD and Δ^9^-THC that were generated by synthesis. Both show a peak at nominal *m*/*z* 628, consistent with 2M^+•^ (where M = *m*/*z* 314). These results indicate that the peak at *m*/*z* 629 observed for hemp samples by DART-HRMS, consistent with [2M + H]^+^, is a gas-phase adduct generated during DART-HRMS analysis and represents the corresponding nonproton-bound dimer in FD-MS experiments. Its detection is characteristic of samples containing relatively high levels of CBD (e.g., hemp).

## 4 Conclusions and Implications for the Structural Identity of *m*/*z* 629

In positive-ion mode, various types of ions are detected by DART-HRMS for different classes of molecules, including [M + H]^+^, [M + NH_4_]^+^, [M + M + H]^+^ or [2M + H]^+^, [2M + NH_4_]^+^, [M + O + H]^+^, [2­(M – 2H) + H]^+^, and [M – H_2_O + H]^+^, to name a few.^7,8,33–36^ As demonstrated here, protonated dimers observed by DART-HRMS analysis can take the form of [2M + H]^+^ (such as CBDH^+^••CBD or Δ^9^-THCH^+^••Δ^9^-THC) or [M + M’ + H]^+^ (such as CBDH^+^••Δ^9^-THC or Δ^9^-THCH^+^••CBD). However, the structural features of the detected dimer ion are dependent on several factors. For example, the concentration of the analyte of interest present in the sample can affect the intensity of the peaks of protonated dimer complexes.^36–39^ The basis for this is that the generation of a protonated dimer signal involves two molecules (e.g., CBD and THC, or CBD and CBC, etc.) rather than just one.^39^ Another factor that contributes to the detection of dimers involves the level of fragmentation that the molecules undergo. The intensity of dimer signals typically decreases as the orifice 1 voltage is increased (because further fragmentation is induced).^34^ Therefore, the observation of protonated dimers comprised of two precursor molecules under collision-induced dissociation conditions (e.g., at an orifice 1 voltage of 90 V) would be unlikely. The mass detector voltage also determines whether or not dimers are detected. In our study, it was discovered that at a detector voltage of 2000 V, dimers were not detected, but at a voltage of 2100 V, they were readily observed due to the increased signal reaching the detector. A fourth factor involves mass accuracy. For example, if the experimentally determined mass of a protonated dimer deviates too far from the calculated mass, then it can be difficult to make a tentative identification. Factors that can contribute to such occurrences include: (1) low peak intensity; (2) poor resolution; and (3) the observation of shoulders on the peaks of interest that, when centroided, cause a shift in the mass.^35^

As previously described, this computational study was prompted by interest in the structural identity of the *m*/*z* value revealed to be diagnostic for differentiating between hemp and marijuana varieties of *C. sativa* using DART-HRMS (i.e., nominal *m*/*z* 629). Since the high-resolution mass observed corresponded to a protonated dimer of a cannabinoid with the formula [C_21_H_30_O_2_ + H]^+^, and because analyte protonated dimers are routinely observed in analyses by DART-HRMS, it was surmised that *m*/*z* 629 corresponded to [2 C_21_H_30_O_2_ + H]^+^. However, since: the monomer formula corresponds to multiple cannabinoid isomers (Δ^8^-THC; Δ^9^-THC; and CBC, CBT, and CBD); all of these isomers exist (albeit at characteristically different concentrations) in both hemp and marijuana; and both protonated homo- and heterodimer adducts are possible, the challenge lay in determining which of these was responsible for *m*/*z* 629 under the DART-HRMS analysis conditions used, and why it was only observed for hemp. The measurements were conducted at a DART gas temperature set at 350 °C, which is equivalent to 623.15 K. According to Figure 9, it is CBDH^+^-2••CBD (navy line) dimer arrangements are the most abundant at this temperature. Furthermore, at 623.15 K, the population of Δ^9^-THCH^+^-6••CBD is significantly less than that of the CBDH^+^-2••CBD dimer (purple line). Thus, based on our calculations, the dimer structure that is primarily responsible for the appearance of *m*/*z* 629 in the DART mass spectrum of hemp under the analysis conditions is CBDH^+^-2••CBD (Figure 2).

The conclusions drawn in this work are further supported by the results observed in our previous study involving hemp and marijuana plant materials. A peak at *m*/*z* 629 was detected in all 41 of the hemp samples,^6^ which were reported by the vendors to contain large amounts of CBD and ≤0.3% Δ^9^-THC. This was not the case when marijuana plant materials were analyzed by DART-HRMS. Although a peak at *m*/*z* 629 was observed in DART-HRMS analysis of a Δ^9^-THC standard in the current study, it was not observed in the DART-HR mass spectra of 33 samples that were reported by the vendors to be marijuana by virtue of containing >0.3% Δ^9^-THC. It has been previously demonstrated that the complexity of a matrix can interfere with the detection of *Cannabis*-related molecules.^4^ This is because when Δ^9^-THC is present within a complex matrix such as *C. sativa* plant materials or derived products, there are hundreds of other molecules in the sample. This increases the number of peaks observed in its spectrum, which can obscure lower-intensity peaks (e.g., dimers). Therefore, due to the complexity of the *Cannabis* plant material and the lower stability of Δ^9^-THC••Δ^9^-THC dimers at the 350 °C temperature of the DART-HRMS measurement, it can be inferred that the peak at *m*/*z* 629 determined to be diagnostic for differentiating between hemp and marijuana plant materials was likely due to the presence of the CBDH^+^••CBD dimer.

Because other cannabinoids share the same molecular formula as Δ^9^-THC and CBD (e.g., CBC, Δ^8^-THC and CBT), the formation of the respective protonated dimer complexes was also investigated in this work. The computational results suggest that at the DART gas temperature of 623.15 K, the population of Δ^8^-THCH^+^-6••CBC, CBCH^+^-1••CBC, CBDH^+^-2••CBC are 9%, 0.3%, and 4.5% respectively. This result indicates that these three proton-bound dimers make only negligible contributions if at all to the overall population of *m*/*z* 629 masses. Trace amounts of CBC and Δ^8^-THC present in the cannabinoid sample were found to participate in dimer formation through Δ^8^-THCH^+^-6••CBC, CBCH^+^-1••CBC, and CBDH^+^-2••CBC.

## Supplementary Material



## Data Availability

The Gaussian output files for all the reactants, protonated monomers, and protonated dimers involved in the quantum chemical calculations are available online (10.5281/zenodo.18774171).

## Notes

Figures S1–S17 present the numbering of the possible protonation sites of CBD, Δ^9^-THC, CBC, Δ^8^-THC, and CBT, as well as the optimized geometries of protonated monomers and protonated homo- and heterodimers of CBD, Δ^9^-THC, CBC, Δ^8^-THC, and CBT, along with their binding energies; Figure S18 shows the thermometer positions at which the DART gas temperature was measured and the respective values; Figure S19 presents FD-MS spectra of Δ^9^-THC and CBD reference standards; Table S1 specifically provides the enthalpies of the proton-bound homo- and heterodimers of the cannabinoids studied in this work (PDF)
