# Supplementary material for: Investigating the Protonated Cannabinoid Dimer Detected in the Forensic Analysis of Cannabis by DART-MS: A Combined Mass Spectrometry and Computational Study
Source: J Am Soc Mass Spectrom. 2026 Mar 21;37(4):919–33. doi: 10.1021/jasms.5c00433 (PMC13047696; doi:10.1021/jasms.5c00433)
Supplement: Supplementary file 1 [file js5c00433_si_001.pdf]

Supporting Information

Investigating the Protonated Cannabinoid Dimer Detected in the Forensic Analysis of *Cannabis* by DART-MS: A Combined Mass Spectrometry and Computational Study

Parandaman Arathala[a], Benedetta Garosi[a], Megan I. Chambers[b], Robert B. Cody[c] and Rabi A. Musah[a, b, *]

[a]*Department of Chemistry, Louisiana State University, Baton Rouge, Louisiana 70803, USA*

[b]*Department of Chemistry, University at Albany, State University of New York, 1400 Washington Avenue, Albany, NY 12222, USA*

[c]*JEOL USA Inc., 11 Dearborn Road, Peabody, MA, 01960, USA*

*Corresponding author: rmusah@lsu.edu

This section contains: Figures S1–S17 presenting the numbering of the possible protonation sites of CBD, Δ^9-THC, CBC, Δ^8-THC, and CBT, as well as the optimized geometries of protonated monomers and protonated homo- and heterodimers of CBD, Δ^9-THC, CBC, Δ^8-THC, and CBT, along with their binding energies; Figure S18 shows the thermometer positions at which the DART gas temperature was measured and the corresponding values; Figure S19 presents FD-MS spectra of CBD and Δ^9-THC reference standards; Table S1 which provides the enthalpies of the proton-bound homo- and heterodimers of the cannabinoids studied in this work.

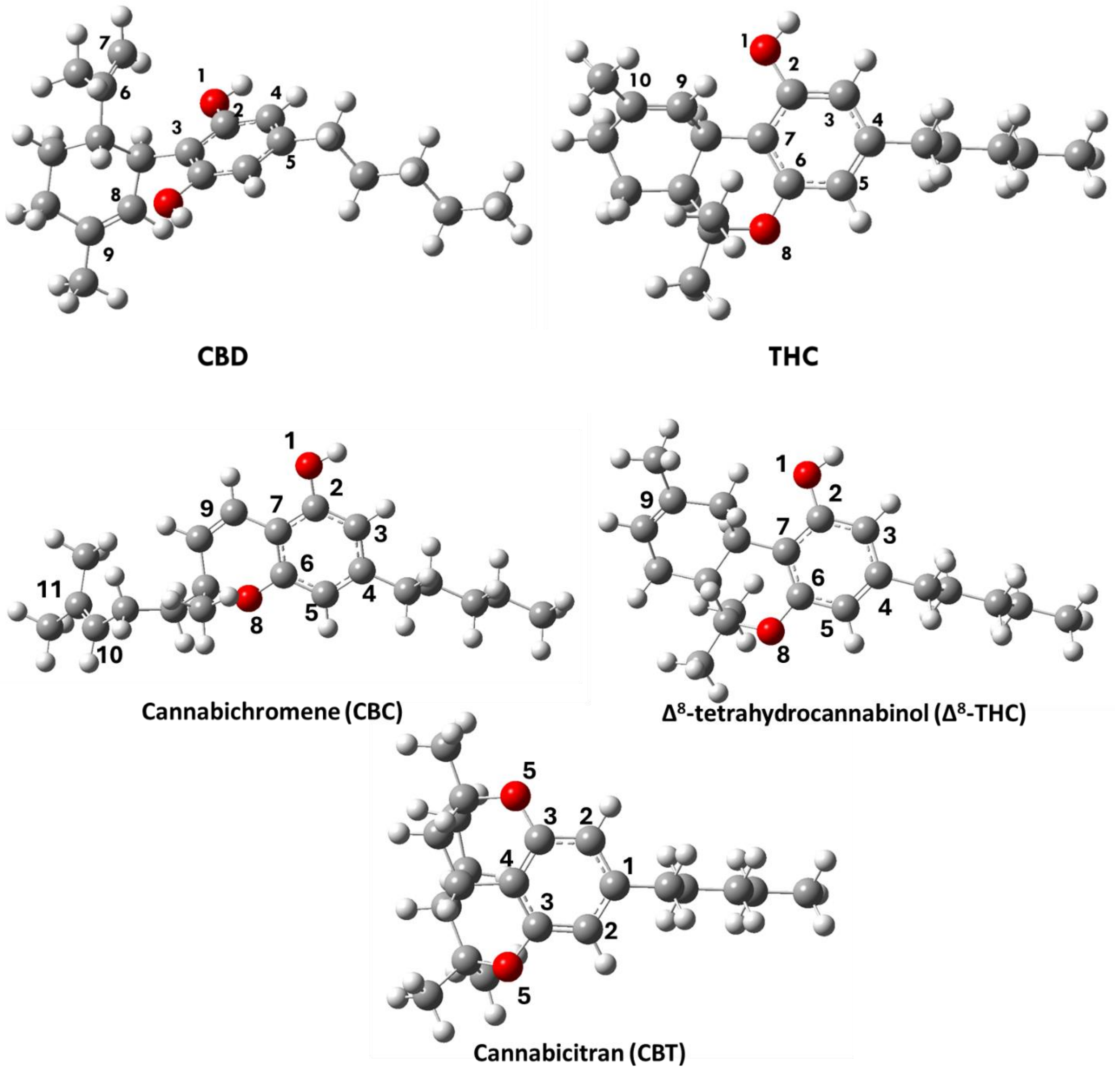

Figure S1. Numbering of the various possible protonation sites in the geometries of CBD, Δ^9-THC, CBC, Δ^8-THC and CBT, optimized at the M06-2X/6-311+G(2d,2p) level of theory. Carbon, hydrogen, and oxygen atoms are shown in grey, white, and red, respectively.

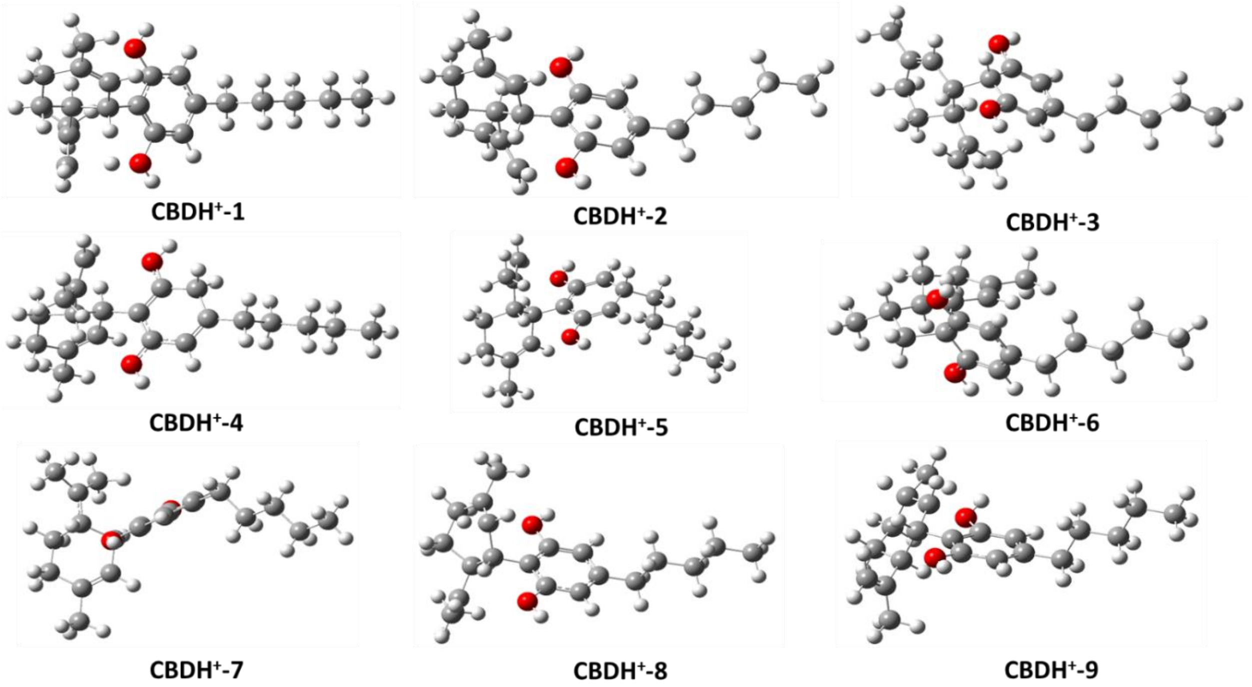

Figure S2. The M06-2X/6-311++G(2d,2p) level optimized geometries of the protonated monomers of CBD. The C, H, and O-atoms are represented with grey, white and red colors, respectively.

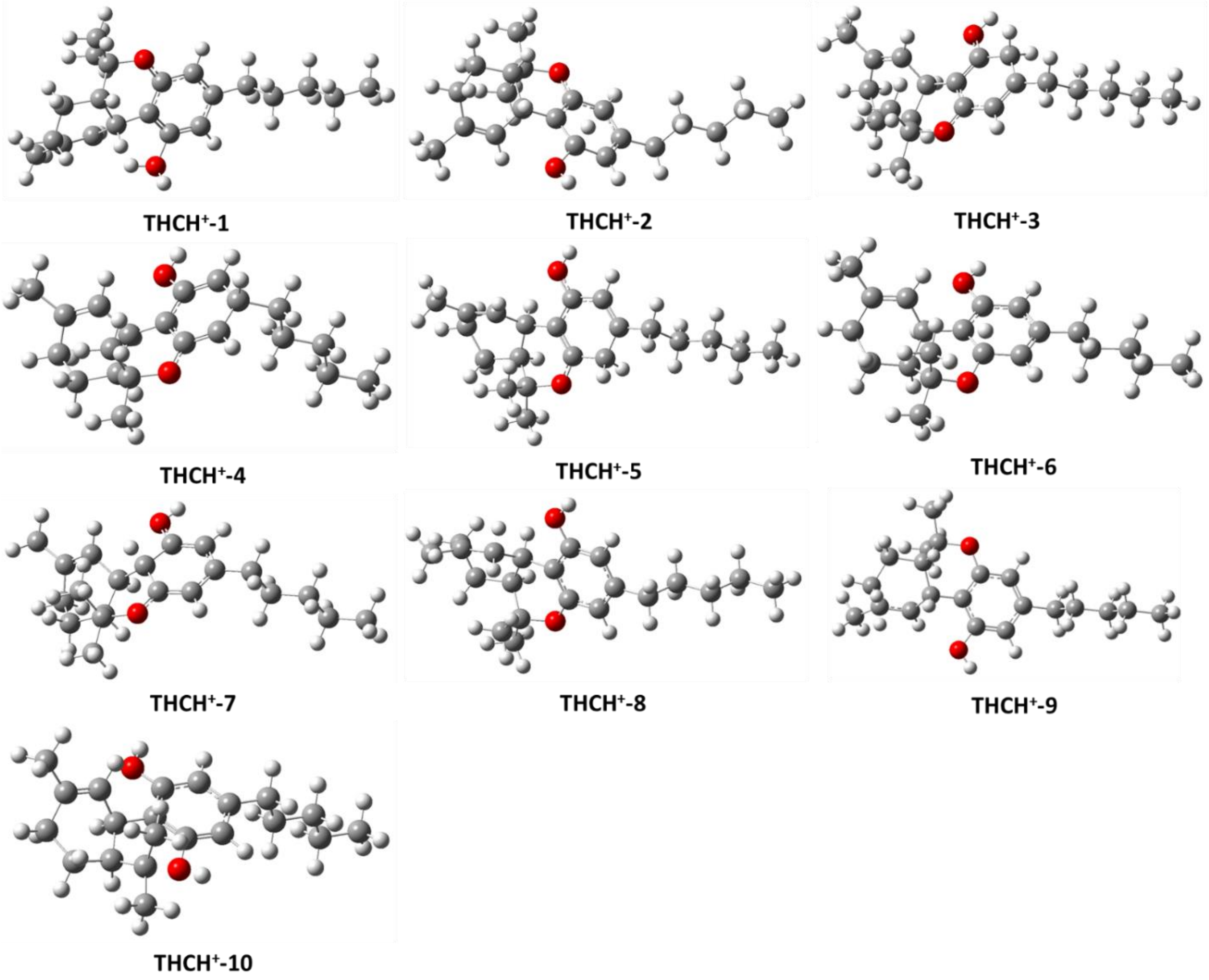

Figure S3. The M06-2X/6-311++G(2d,2p) level optimized geometries of the protonated monomers of Δ^9-THC. The C, H, and O-atoms are represented with grey, white and red colors, respectively.

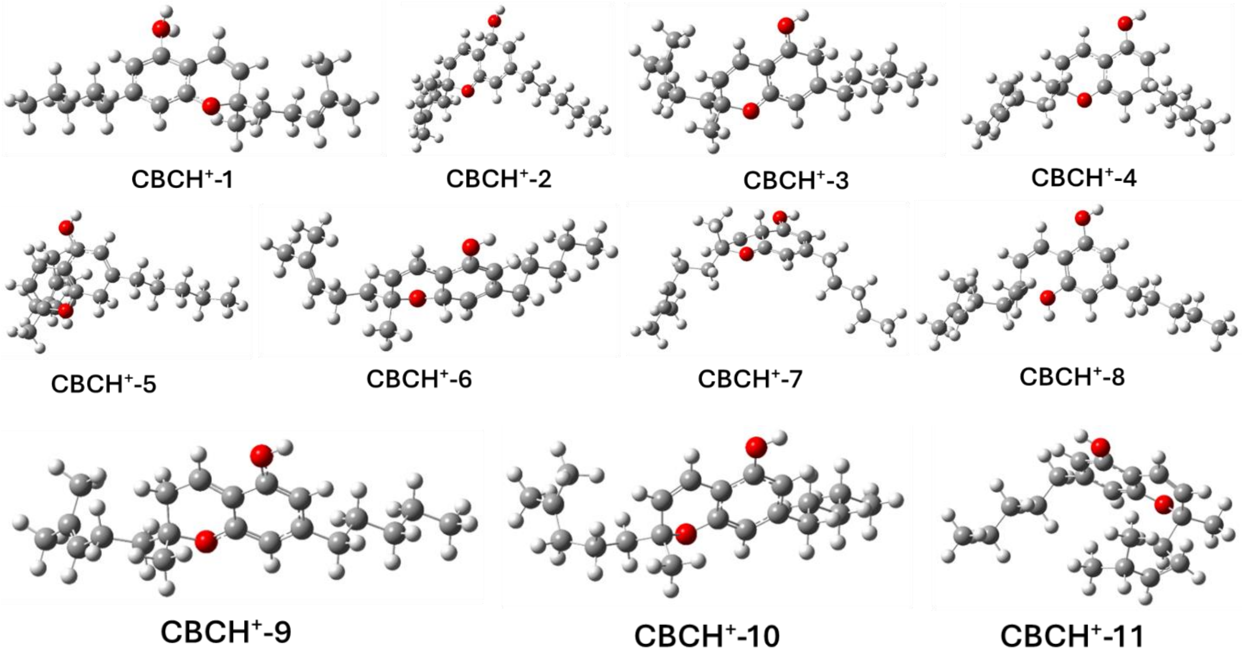

Figure S4. The M06-2X/6-311++G(2d,2p) level optimized geometries of the protonated monomers of CBC. The C, H, and O-atoms are represented with grey, white and red colors, respectively.

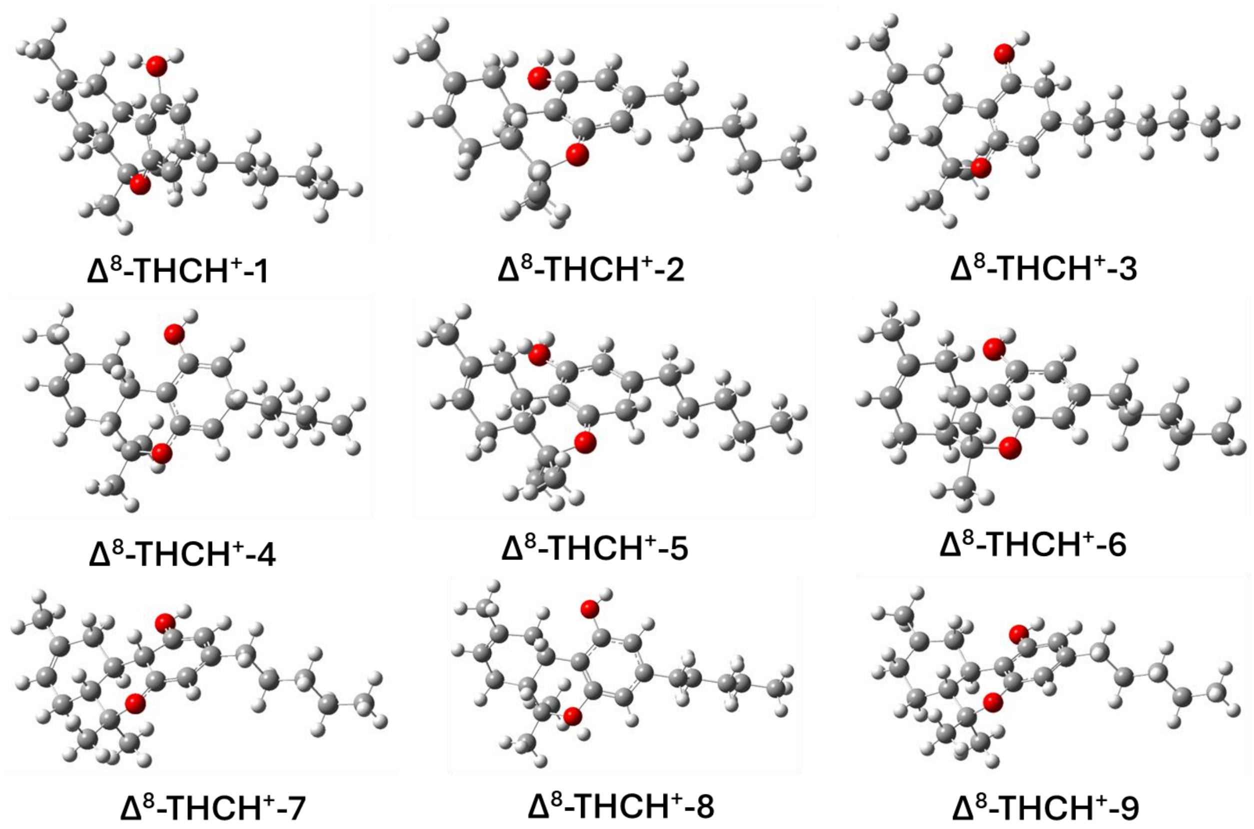

Figure S5. The M06-2X/6-311++G(2d,2p) level optimized geometries of the protonated monomers of Δ^8-THC. The C, H, and O-atoms are represented with grey, white and red colors, respectively.

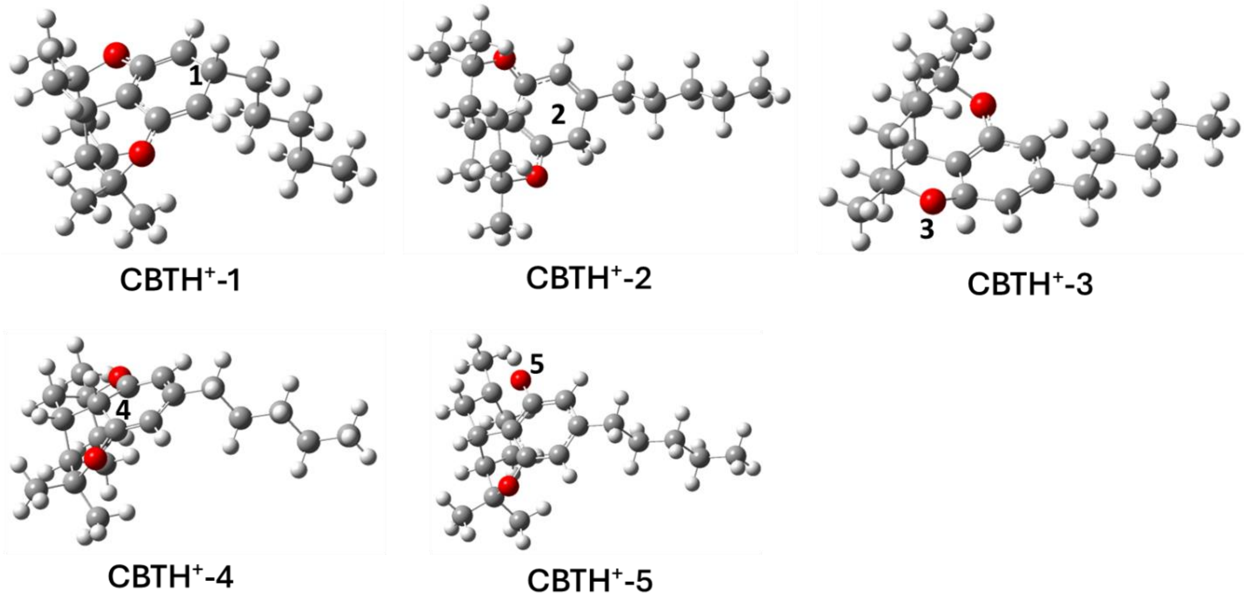

Figure S6. The M06-2X/6-311++G(2d,2p) level optimized geometries of the protonated monomers of CBT. The C, H, and O-atoms are represented with grey, white and red colors, respectively.

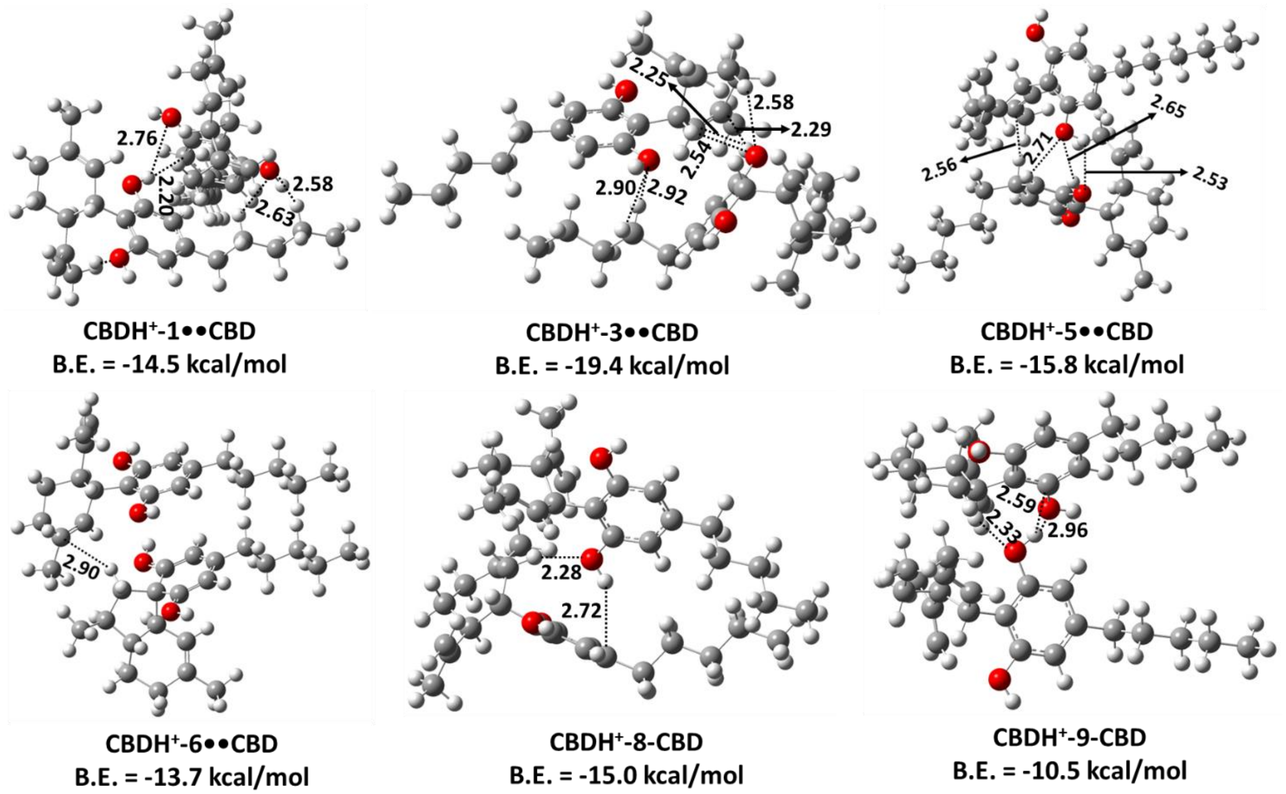

Figure S7. Less stable structures of the various possible CBD proton-bound dimers obtained at the M06-2X/6-311++G(2d,2p) level of theory. Bond lengths are reported in units of Angstroms (Å). The binding energies (B.E.) obtained at the M06-2X/6-311++G(3df,3pd)//M06-2X/6-311++G(2d,2p) level. The C, H, and O-atoms are represented with grey, white and red colors, respectively.

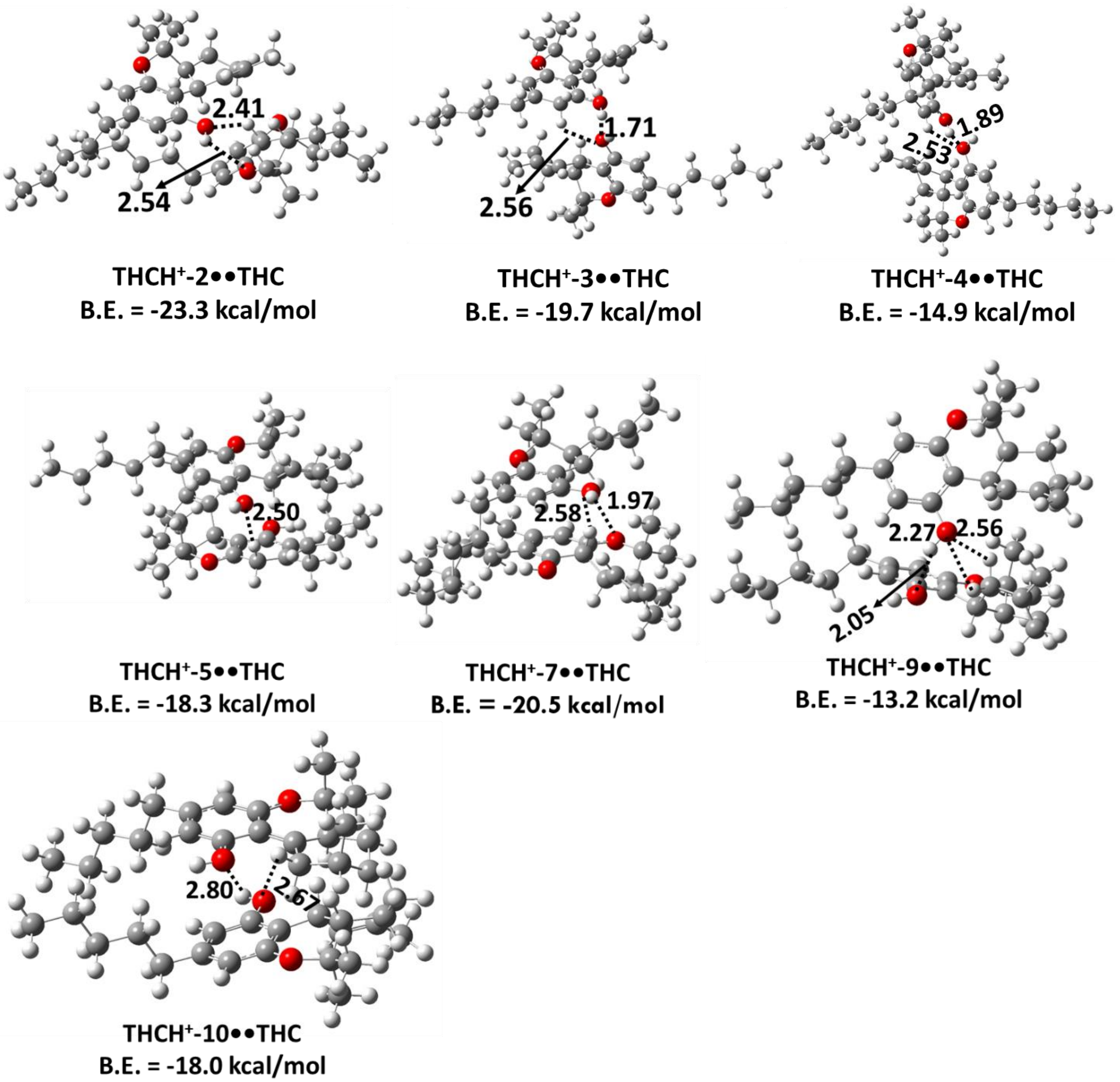

Figure S8. Less stable structures of the various possible Δ^9-THC proton-bound dimers formed by the interaction of protonated Δ^9-THC with the -OH group of neutral Δ^9-THC, optimized at the M06-2X/6-311++G(2d,2p) level of theory. Bond lengths are reported in units of Angstroms (Å). The binding energies (B.E.) obtained at the M06-2X/6-311++G(3df,3pd)//M06-2X/6-311++G(2d,2p) level. The C, H, and O-atoms are represented with grey, white and red colors, respectively.

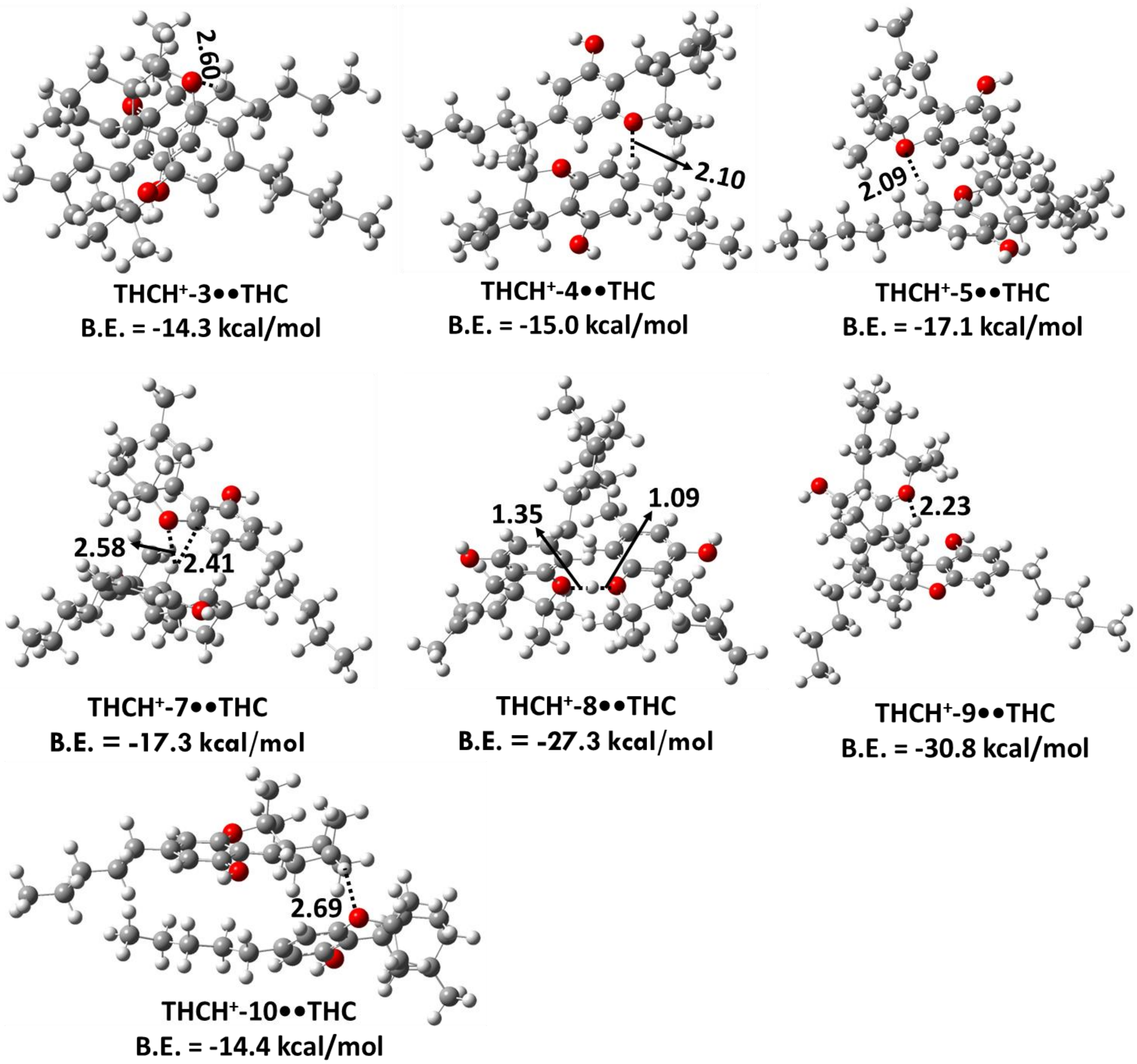

Figure S9. Less stable structures of the various possible Δ^9-THC proton-bound dimers formed by the interaction of protonated Δ^9-THC with the O-atom of pyran ring in neutral Δ^9-THC, optimized at the M06-2X/6-311++G(2d,2p) level of theory. Bond lengths are reported in units of Angstroms (Å). The binding energies (B.E.) obtained at the M06-2X/6-311++G(3df,3pd)//M06-2X/6-311++G(2d,2p) level. The C, H, and O-atoms are represented with grey, white and red colors, respectively.

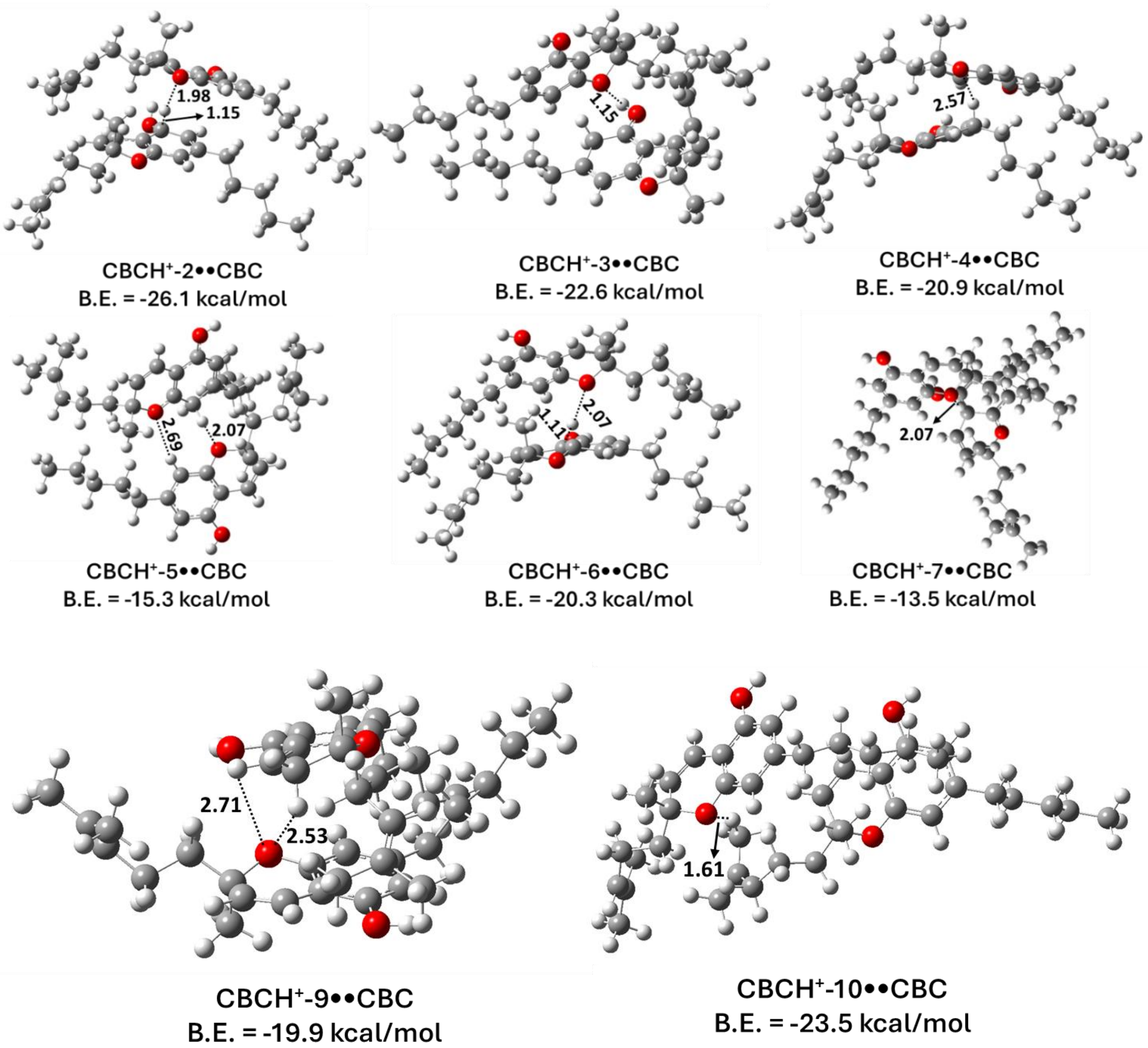

Figure S10. Less stable structures of the possible $CBCH^{+}$••CBC proton-bound homodimers were obtained, where protonated CBC interacts with the O-atom of the pyran ring in neutral CBC, at the M06-2X/6-311++G(2d,2p) level of theory. Bond lengths are reported in units of Angstroms (Å). The binding energies (B.E.) obtained at the M06-2X/6-311++G(3df,3pd)//M06-2X/6-311++G(2d,2p) level. The C, H, and O-atoms are represented with grey, white and red colors, respectively.

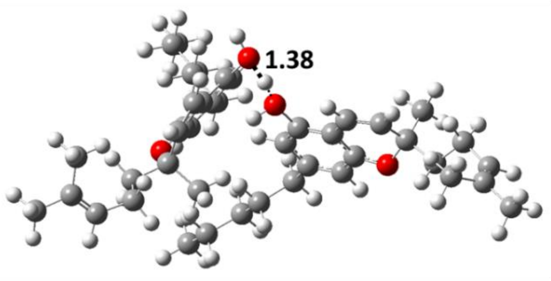

CBCH+-1••CBC
B.E. = -32.9 kcal/mol

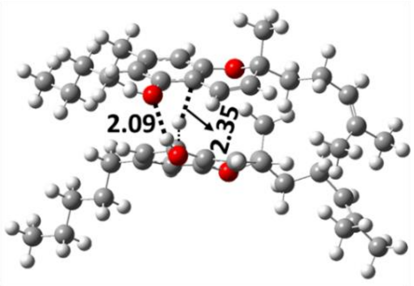

CBCH+-2••CBC
B.E. = -25.2 kcal/mol

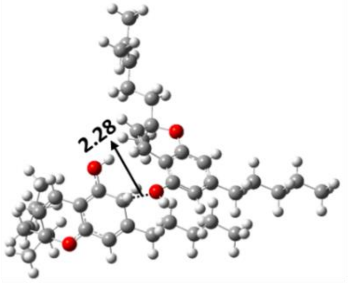

CBCH+-3••CBC
B.E. = -21.0 kcal/mol

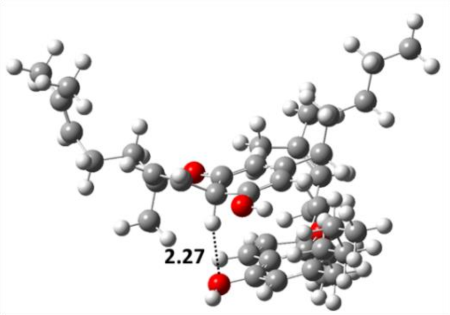

CBCH+-4••CBC
B.E. = -13.2 kcal/mol

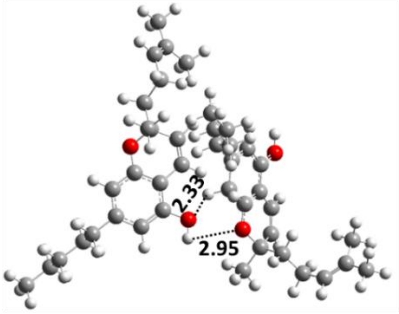

CBCH+-5••CBC
B.E. = -10.4 kcal/mol

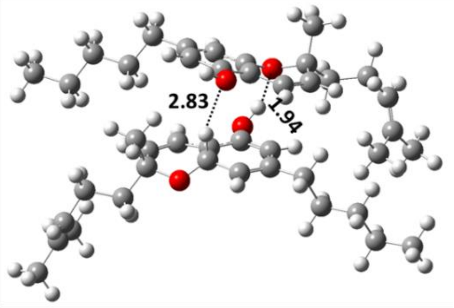

CBCH+-6••CBC
B.E. = -25.1 kcal/mol

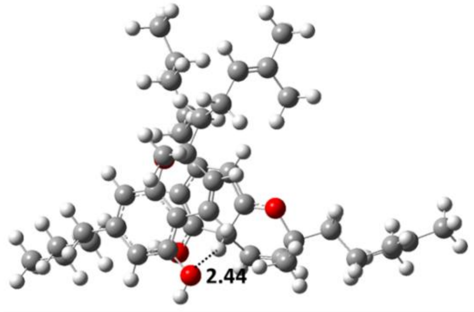

CBCH+-7••CBC
B.E. = -20.8 kcal/mol

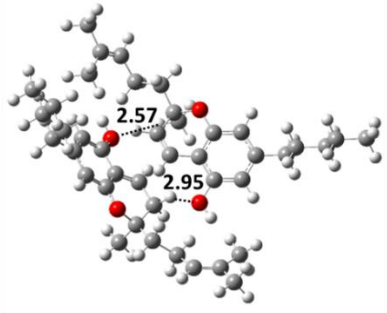

CBCH+-8••CBC
B.E. = -15.7 kcal/mol

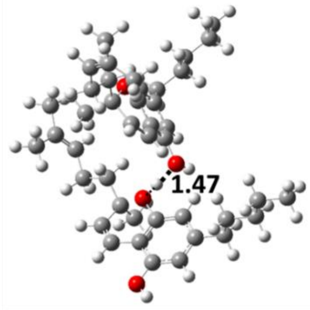

CBCH+-9••CBC
B.E. = -25.4 kcal/mol

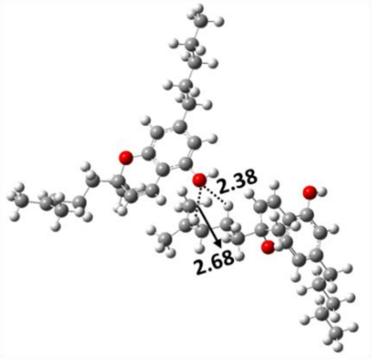

CBCH+-10••CBC
B.E. = -16.1 kcal/mol

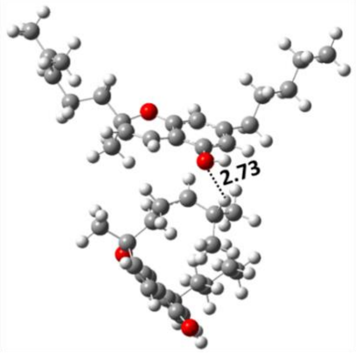

CBCH+-11••CBC
B.E. = -16.1 kcal/mol

Figure S11. Various possible $CBCH^{+}$••CBC proton-bound homodimers were obtained, where protonated CBC interacts with the OH-moiety of neutral CBC, at the M06-2X/6-311++G(2d,2p) level of theory. Bond lengths are reported in units of Angstroms (Å). The binding energies (B.E.) obtained at the M06-2X/6-311++G(3df,3pd)//M06-2X/6-311++G(2d,2p) level. The C, H, and O-atoms are represented with grey, white and red colors, respectively.

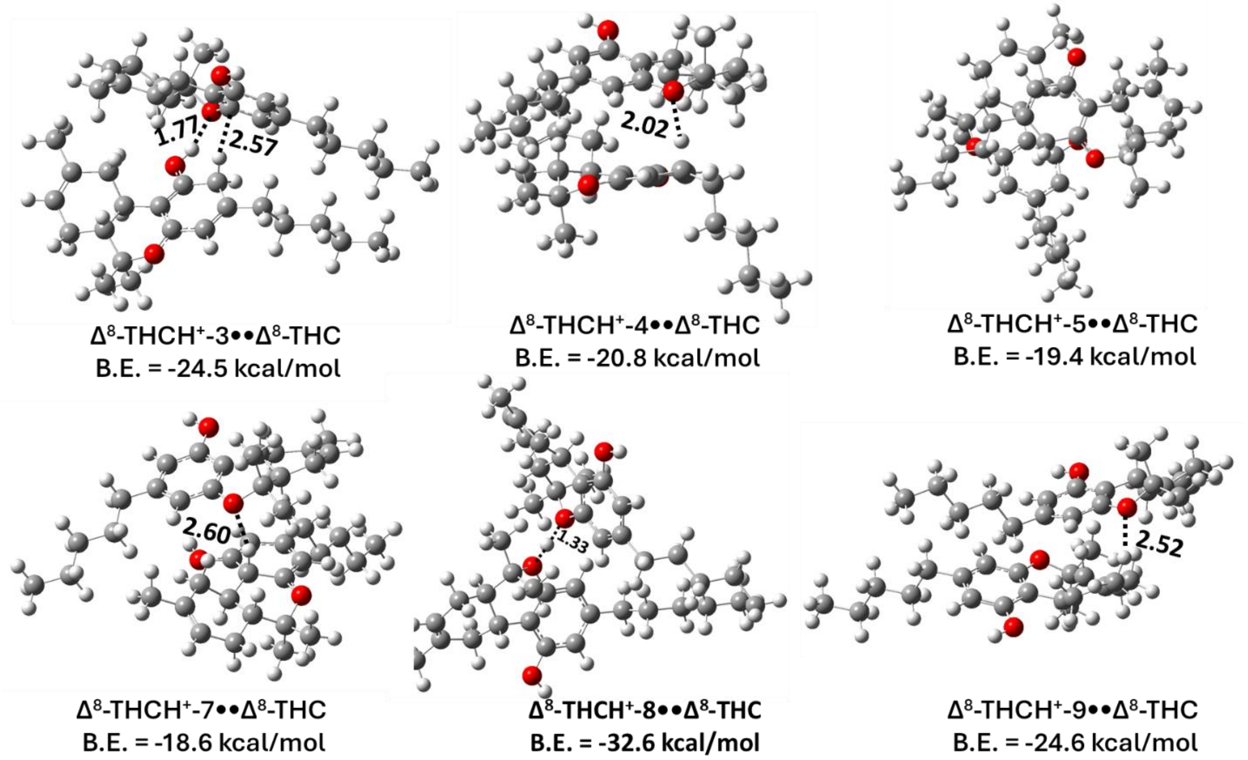

Figure S12. Less stable structures of proton-bound homodimers of Δ^8-THC formed via protonated Δ^8-THC interacting with the O-atom of the pyran ring in neutral Δ^8-THC, optimized at the M06-2X/6-311++G(2d,2p) level of theory. Bond lengths are reported in Å. Binding energies (B.E.) were calculated at the M06-2X/6-311++G(3df,3pd)//M06-2X/6-311++G(2d,2p) level of theory. Carbon, hydrogen, and oxygen atoms are represented in grey, white, and red, respectively.

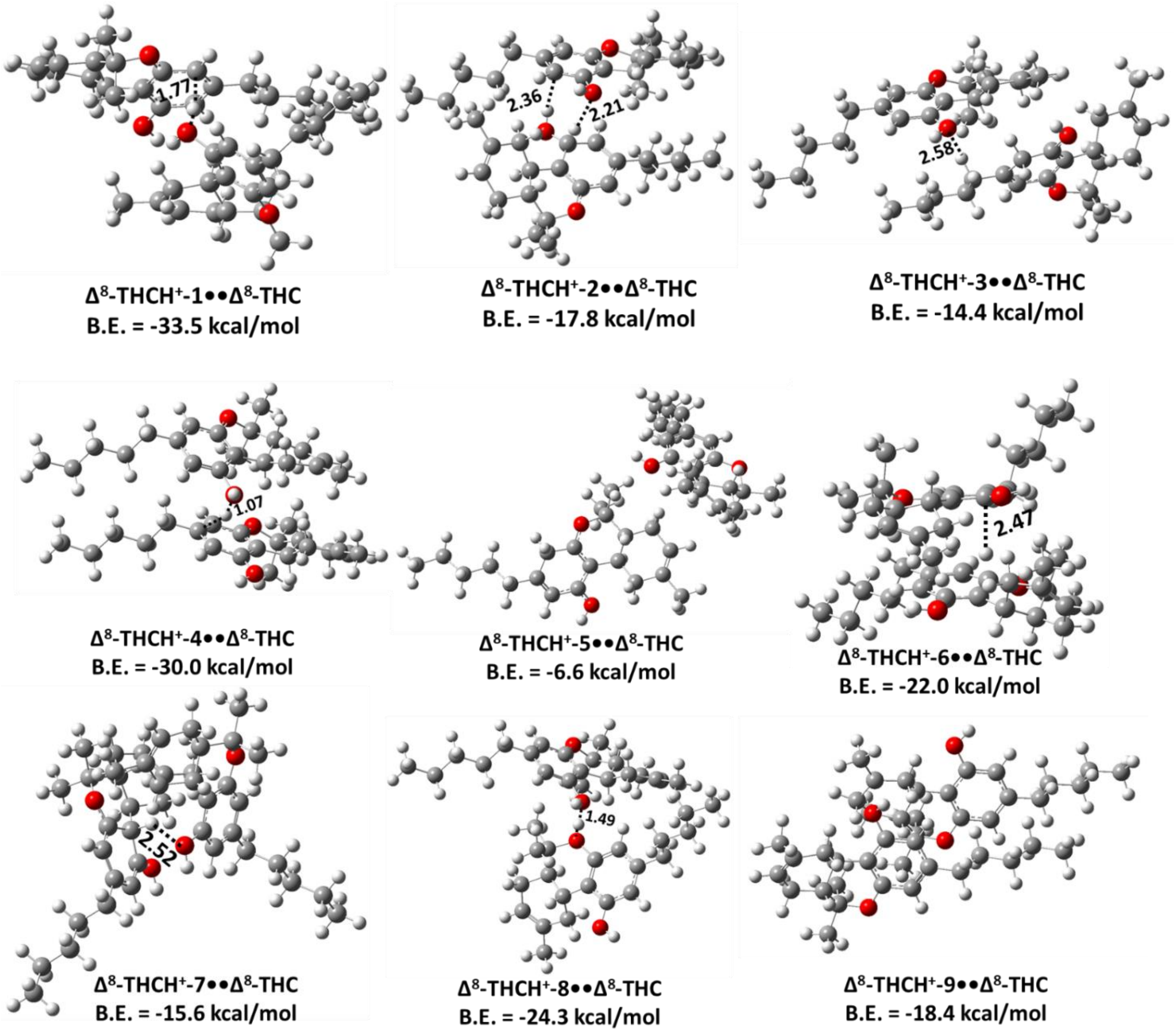

Figure S13. Various possible proton-bound homodimers of Δ^8-THC formed via protonated Δ^8-THC interacts with the OH-moiety of neutral Δ^8-THC, optimized at the M06-2X/6-311++G(2d,2p) level of theory. Bond lengths are reported in units of Angstroms (Å). The binding energies (B.E.)obtained at the M06-2X/6-311++G(3df,3pd)//M06-2X/6-311++G(2d,2p) level. The C, H, and O-atoms are represented with grey, white and red colors, respectively.

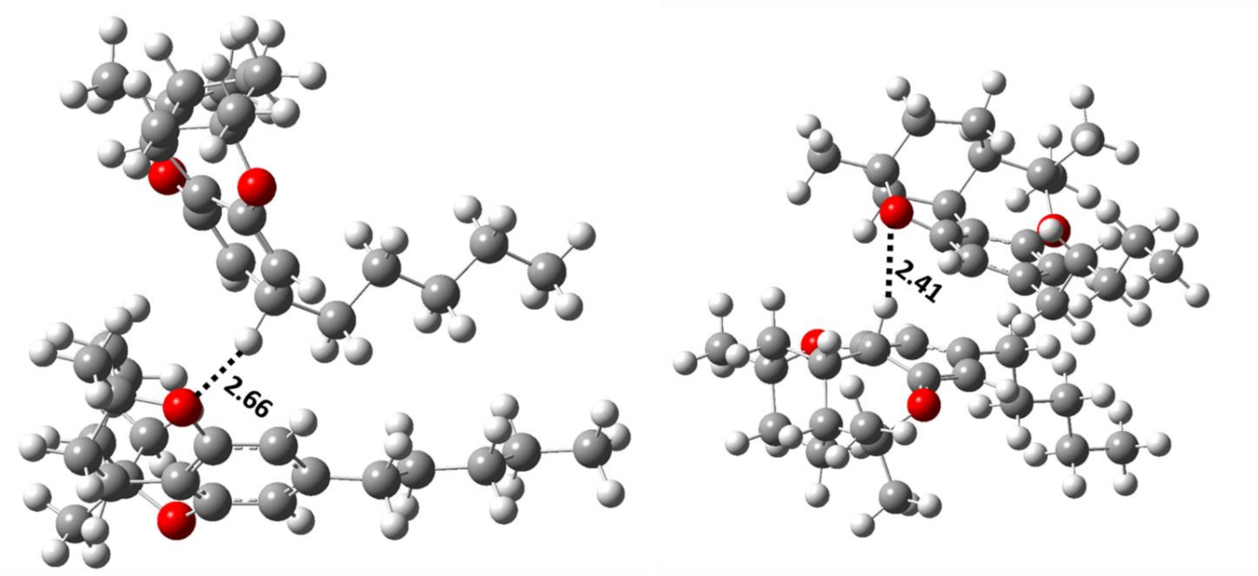

CBTH$^+$-1••CBT
B.E. = -13.0 kcal/mol

CBTH$^+$-4••CBT
B.E. = -17.6 kcal/mol

Figure S14. Less stable structures of proton-bound homodimers of CBT optimized at the M06-2X/6-311++G(2d,2p) level of theory. Bond lengths are reported in Å. Binding energies (B.E.) were calculated at the M06-2X/6-311++G(3df,3pd)//M06-2X/6-311++G(2d,2p) level of theory. Carbon, hydrogen, and oxygen atoms are represented in grey, white, and red, respectively.

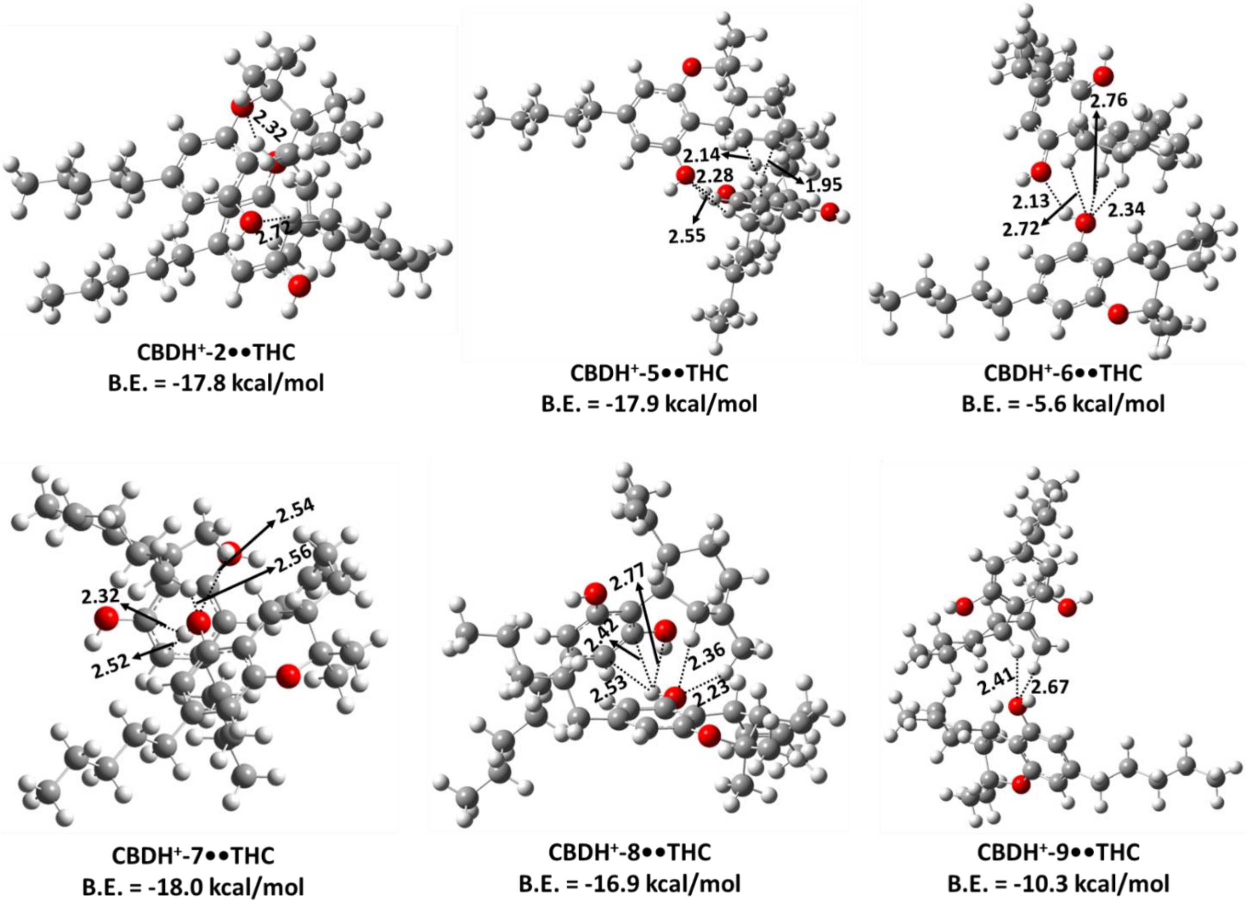

Figure S15. Less stable structures of the various possible CBDH$^+$••Δ^9-THC proton-bound dimers formed by the interaction of protonated CBD with the -OH group of neutral Δ^9-THC, optimized at the M06-2X/6-311++G(2d,2p) level of theory. Bond lengths are reported in units of Angstroms (Å). The binding energies (B.E.) obtained at the M06-2X/6-311++G(3df,3pd)//M06-2X/6-311++G(2d,2p) level. The C, H, and O-atoms are represented with grey, white and red colors, respectively.

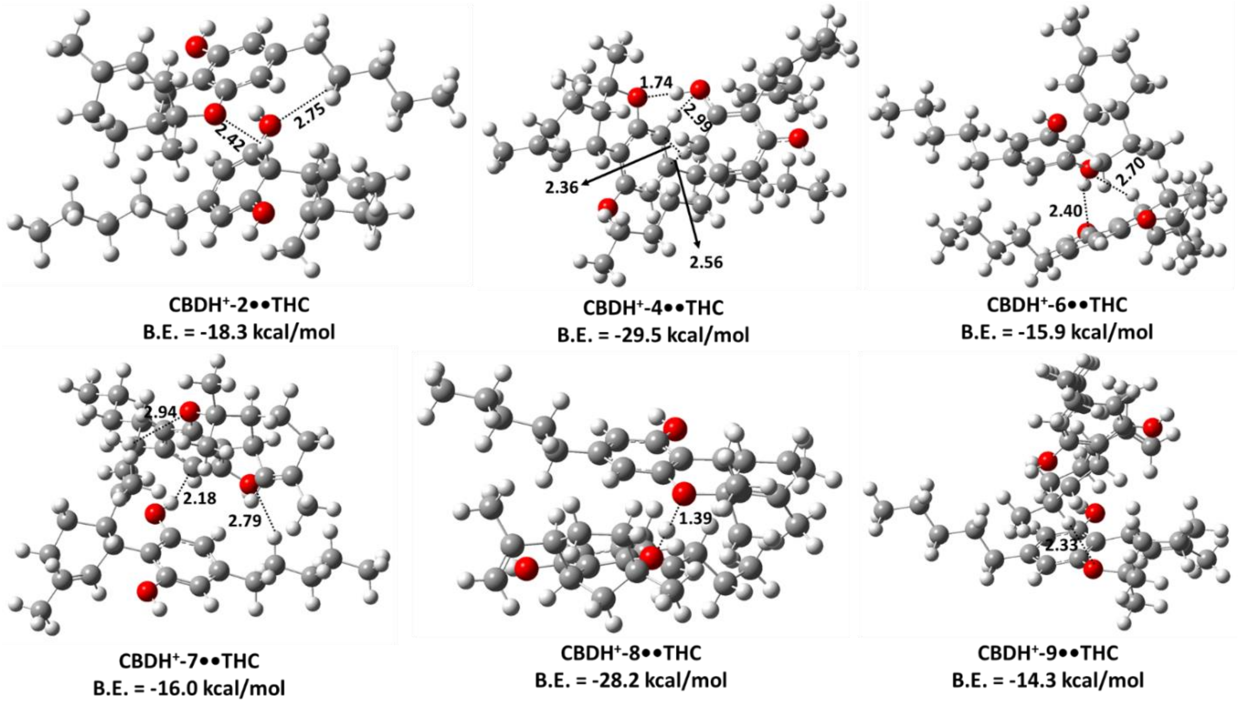

Figure S16. Less stable structures of the various possible CBDH^{+}••Δ^{9}-THC proton-bound dimers formed by the interaction of protonated CBD with the O-atom of pyran ring in neutral Δ^{9}-THC, optimized at the M06-2X/6-311++G(2d,2p) level of theory. Bond lengths are reported in units of Angstroms (Å). The binding energies (B.E.) obtained at the M06-2X/6-311++G(3df,3pd)//M06-2X/6-311++G(2d,2p) level. The C, H, and O-atoms are represented with grey, white and red colors, respectively.

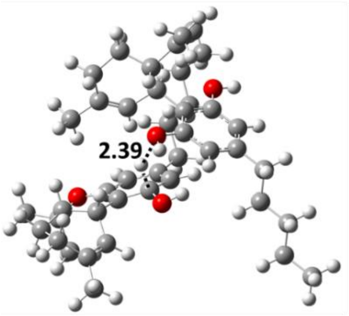

THCH⁺-2••CBD
B.E. = -24.1 kcal/mol

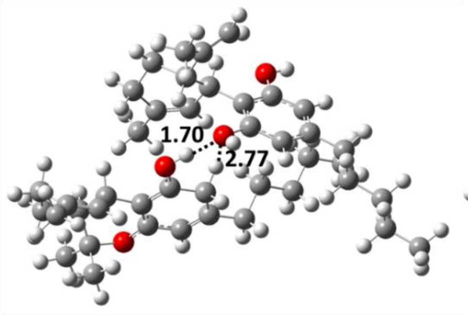

THCH⁺-3••CBD
B.E. = -25.0 kcal/mol

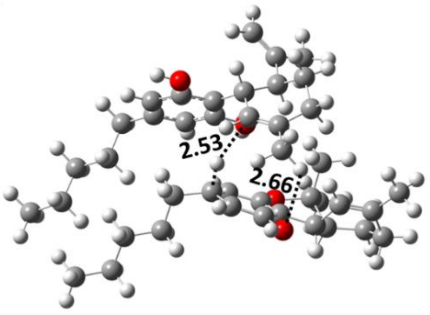

THCH⁺-4••CBD
B.E. = -18.3 kcal/mol

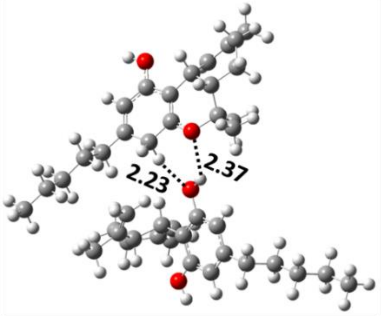

THCH⁺-5••CBD
B.E. = -11.7 kcal/mol

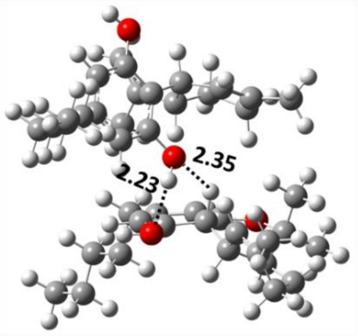

THCH⁺-7••CBD
B.E. = -10.3 kcal/mol

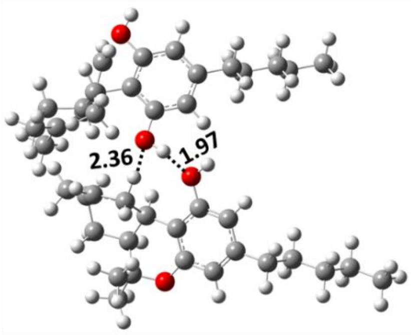

THCH⁺-9••CBD
B.E. = -14.9 kcal/mol

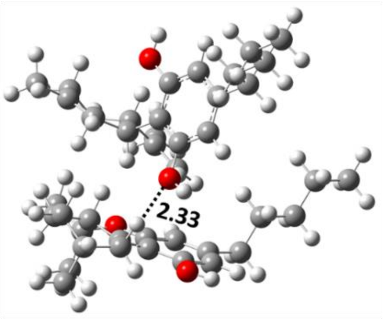

THCH⁺-10••CBD
B.E. = -13.9 kcal/mol

Figure S17. Less stable structures of the various possible Δ^9-THCH$^+$••CBD proton-bound dimers obtained at the M06-2X/6-311++G(2d,2p) level of theory. Bond lengths are reported in units of Angstroms (Å). The binding energies (B.E.) obtained at the M06-2X/6-311++G(3df,3pd)//M06-2X/6-311++G(2d,2p) level. The C, H, and O-atoms are represented with grey, white and red colors, respectively.

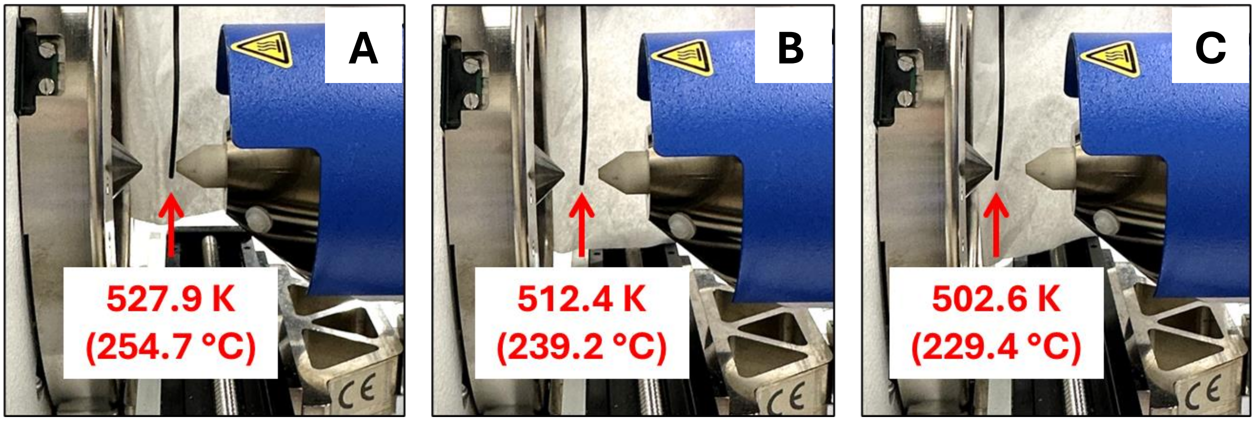

Figure S18. Thermometer positioning between the DART ion source and the mass spectrometer inlet (1 cm apart) at which the metastable helium gas temperature was measured, and the corresponding values reported in Kelvin and degree Celsius. The thermometer probe was positioned approximately 1 mm, 5 mm, and 9 mm from the DART ion source in Panels A, B, and C, respectively.

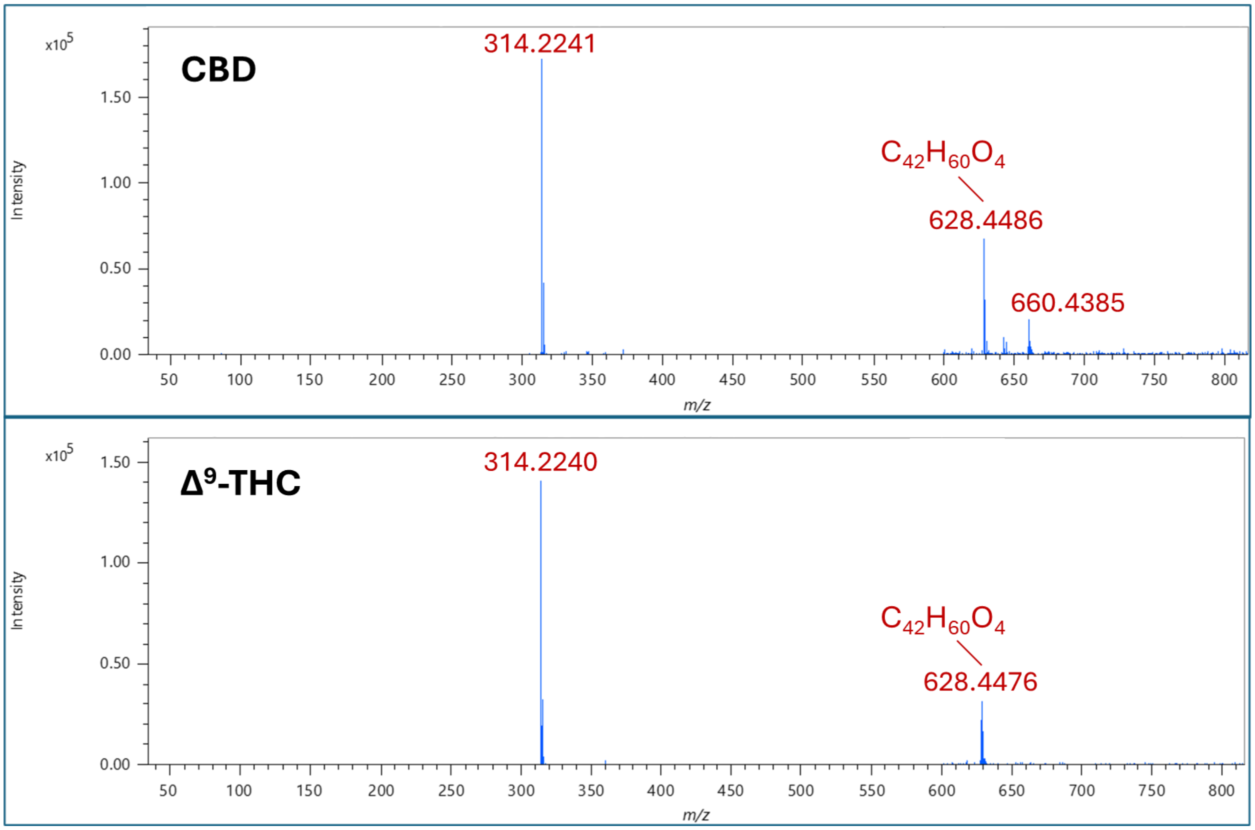

Figure S19. Representative FD-MS spectra for cannabidiol (top) and Δ^9-THC (bottom) certified reference materials. The peak at nominal *m/z* 628 observed in both mass spectra is consistent with the mass of a non-proton-bound dimer with composition $C_{42}H_{58}O_4^+$. If this represented a hemp-specific biomarker, it would not be observed in these synthetic standards. The FD mass spectrum of CBD also exhibits oxidized dimer peaks $C_{42}H_{58}O_5^+$, $C_{42}H_{60}O_5^+$, and $C_{42}H_{60}O_6^+$ (not labeled), attributed to ion-molecule reactions that are specific to CBD. The oxidized dimers are not observed in FD mass spectra of hemp and cannabis (data not shown).

Table S1. Enthalpies of various proton-bound homo- and heterodimers of CBD, Δ^{9}-THC, CBC, Δ^{8}-THC, and CBT at M06-2X/6-311++G(3df,3pd)//M06-2X/6-311++G(2d,2p) level.[a]

Dimer	Enthalpy (kcal/mol)	Dimer	Enthalpy (kcal/mol)
CBDH^{+}-2••CBD	-60.4	CBDH^{+}-1••Δ^{9}-THC (OH)	-26.9
CBDH^{+}-4••CBD	-24.7	CBDH^{+}-3••Δ^{9}-THC (OH)	-51.7
CBDH^{+}-7••CBD	-23.5	CBDH^{+}-4••Δ^{9}-THC (OH)	-21.9
Δ^{9}-THCH^{+}-1••Δ^{9}-THC (OH)	-32.8	CBDH^{+}-1••Δ^{9}-THC (O)	-36.0
Δ^{9}-THCH^{+}-6••Δ^{9}-THC (OH)	-48.2	CBDH^{+}-3••Δ^{9}-THC (O)	-51.2
Δ^{9}-THCH^{+}-8••Δ^{9}-THC (OH)	-26.9	CBDH^{+}-5••THC (O)	-39.4
Δ^{9}-THCH^{+}-1••Δ^{9}-THC (O)	-40.8	Δ^{9}-THCH^{+}-1••CBD	-38.7
THCH^{+}-2••THC (O)	-61.0	Δ^{9}-THCH^{+}-6••CBD	-55.3
THCH^{+}-6••THC (O)	-54.5	Δ^{9}-THCH^{+}-8••CBD	-28.6
CBCH^{+}-1••CBC (O)	-41.8	Δ^{8}-THCH^{+}-1••Δ^{8}-THC	-41.5
CBCH^{+}-8••CBC (O)	-33.9	Δ^{8}-THCH^{+}-2••Δ^{8}-THC	-45.1
CBCH^{+}-11••CBC (O)	-36.6	Δ^{8}-THCH^{+}-6••Δ^{8}-THC	-49.9
CBTH^{+}-2••CBT	-18.2	CBDH^{+}-2••CBC	-58.6
CBTH^{+}-3••CBT	-43.8	CBDH^{+}-2••Δ^{8}-THC	-23.4
CBTH^{+}-5••CBT	-21.8	CBDH^{+}-5••CBT	-55.7
Δ^{9}-THCH^{+}-3••CBC	-24.2	CBCH^{+}-1••CBD	-43.1
Δ^{9}-THCH^{+}-3••Δ^{8}-THC	-44.7	CBCH^{+}-1••Δ^{9}-THC	-41.4
Δ^{9}-THCH^{+}-3••CBT	-45.9	CBCH^{+}-1••Δ^{8}-THC	-43.7
Δ^{8}-THCH^{+}-6••CBD	-51.8	CBCH^{+}-1••CBT	-42.4
Δ^{8}-THCH^{+}-6••Δ^{9}-THC	-45.9	Δ^{8}-THCH^{+}-6••CBC	-61.3
CBTH^{+}-3••CBD	-49.4	Δ^{8}-THCH^{+}-6••CBT	-52.6
CBTH^{+}-3••Δ^{9}-THC	-44.1	CBTH^{+}-3••Δ^{8}-THC	-43.8
CBTH^{+}-3••CBC	-43.3		

[a]Enthalpies were calculated relative to the energy of the corresponding starting separated monomers.
